# Chromosome-scale genome assembly of *Glycyrrhiza uralensis* revealed metabolic gene cluster centred specialized metabolites biosynthesis

**DOI:** 10.1093/dnares/dsac043

**Published:** 2022-12-20

**Authors:** Amit Rai, Hideki Hirakawa, Megha Rai, Yohei Shimizu, Kenta Shirasawa, Shinji Kikuchi, Hikaru Seki, Mami Yamazaki, Atsushi Toyoda, Sachiko Isobe, Toshiya Muranaka, Kazuki Saito

**Affiliations:** RIKEN Center for Sustainable Resource Science, Yokohama, Japan; Plant Molecular Science Center, Chiba University, Chiba, Japan; Kazusa DNA Research Institute, Kisarazu, Chiba, Japan; Plant Molecular Science Center, Chiba University, Chiba, Japan; Graduate School of Pharmaceutical Sciences, Chiba University, Chiba, Japan; Institute of Advance Academic Research, Chiba University, Chiba, Japan; Graduate School of Pharmaceutical Sciences, Chiba University, Chiba, Japan; Kazusa DNA Research Institute, Kisarazu, Chiba, Japan; Plant Molecular Science Center, Chiba University, Chiba, Japan; Graduate School of Horticulture, Chiba University, Chiba, Japan; RIKEN Center for Sustainable Resource Science, Yokohama, Japan; Department of Biotechnology, Graduate School of Engineering, Osaka University, Suita, Japan; Plant Molecular Science Center, Chiba University, Chiba, Japan; Graduate School of Pharmaceutical Sciences, Chiba University, Chiba, Japan; Advanced Genomics Center, National Institute of Genetics, Mishima, Shizuoka, Japan; Kazusa DNA Research Institute, Kisarazu, Chiba, Japan; RIKEN Center for Sustainable Resource Science, Yokohama, Japan; Department of Biotechnology, Graduate School of Engineering, Osaka University, Suita, Japan; RIKEN Center for Sustainable Resource Science, Yokohama, Japan; Plant Molecular Science Center, Chiba University, Chiba, Japan

**Keywords:** *Glycyrrhiza uralensis*, plant gene cluster, glycyrrhizin biosynthesis, saponins, genome assembly

## Abstract

A high-quality genome assembly is imperative to explore the evolutionary basis of characteristic attributes that define chemotype and provide essential resources for a molecular breeding strategy for enhanced production of medicinal metabolites. Here, using single-molecule high-fidelity (HiFi) sequencing reads, we report chromosome-scale genome assembly for Chinese licorice (*Glycyrrhiza uralensis*), a widely used herbal and natural medicine. The entire genome assembly was achieved in eight chromosomes, with contig and scaffold N50 as 36.02 and 60.2 Mb, respectively. With only 17 assembly gaps and half of the chromosomes having no or one assembly gap, the presented genome assembly is among the best plant genomes to date. Our results showed an advantage of using highly accurate long-read HiFi sequencing data for assembling a highly heterozygous genome including its complexed repeat content. Additionally, our analysis revealed that *G. uralensis* experienced a recent whole-genome duplication at approximately 59.02 million years ago post a gamma (γ) whole-genome triplication event, which contributed to its present chemotype features. The metabolic gene cluster analysis identified 355 gene clusters, which included the entire biosynthesis pathway of glycyrrhizin. The genome assembly and its annotations provide an essential resource for licorice improvement through molecular breeding and the discovery of valuable genes for engineering bioactive components and understanding the evolution of specialized metabolites biosynthesis.

## 1. Introduction

The genus *Glycyrrhiza*, commonly known as licorice, belongs to the Fabaceae family and includes approximately 30 species majorly distributed across Europe, Asia, South America, and North America.^1,2^ Licorice has long been used as a sweetener and an essential component of numerous herbal preparations^3^ and represents one of the world’s most extensively researched medicinal plants.^4^ Experimental and clinical studies using metabolic extracts of licorice have shown to exhibit a broad range of activities, including hypocholesterolaemic and hypoglycaemic,^5^ anxiolytic,^4^ antimicrobial and antiviral,^6^ antitumour,^7^ antiallergic,^8,9^ antidiabetic,^10^ anti-inflammatory,^11,12^ and hepatoprotective activities.^13,14^ It has also been effective against dementia, Alzheimer’s disease, and other neurodegenerative disorders.^15^

Flavonoids (over 300) and triterpene saponins (77) are the principal bioactive constituent of licorice.^16^ Flavonoids accumulated in the licorice are majorly glycosides of liquiritigenin and isoliquiritigenin, including liquiritin, isoliquiritin, and licuraside.^17,18^ Glycyrrhizin represents the major component of triterpene saponins, which represents up to 5% of its dry weight and is 50–150 times sweeter than sucrose.^17,19,20^ For its sweet taste, it is also widely used in food and flavor industries, including confectionery, condiments, chocolate, beer, and drinks.^21^ The medicinal and industrial values of licorice have led to its tremendous global trade volume of $261.62 million in 2019, representing an increase of 367.16% compared with 1994.^22^ To effectively meet the increasing global demand, the sustainable, resilient global production of licorice is required, which can be accelerated by the availability of the high-quality genome assembly of *Glycyrrhiza* species.

The advances in long-read sequencing technologies have encouraged resequencing for previously published ‘draft’ plant genomes, resulting in increasingly improved chromosome-scale plant genomes. Long-read sequencing technologies, including Pacific Biosciences, and Oxford Nanopore, have shown significant improvement in assembly contiguity and have become the drivers for high-quality genome assembly projects. However, the inherent random errors within long-read sequencing technology pose challenges for achieving high genome contiguity for plant genomes with high heterozygosity. Recent advances in high-fidelity (HiFi) long-read Pacbio sequencing offer a great advantage of achieving overlaps within highly repetitive genomic regions and, therefore, can achieve a highly contiguous genome even for a complexed and heterozygous plant genome.^23^ HiFi reads could achieve raw read N50 over 15–20 kb with an accuracy of around 99.8%, which enables overcoming the assembly of highly repetitive centromere and telomere regions.^24^ Previously, whole-genome sequencing for *G. uralensis* was reported using a hybrid assembly approach, resulting in 94.5% of the predicted *G. uralensis* genome size into 12,528 scaffolds with 36.8% genome as repeats and scaffolds N50 as 0.1 Mb.^25^ The importance of the *G. uralensis* plant for its medicinal and industrial application makes it urgent to establish a high-quality chromosome-scale genome resource to facilitate the discovery of the biosynthesis of specialized metabolites and species improvement.

This study reports chromosome-scale genome assembly for *G. uralensis* using HiFi sequencing technologies and a Hi-C-based scaffolding approach. We optimized the assembly parameter to achieve a highly contiguous genome assembly and used a stepwise assembly validation approach to derive a chromosome-scale genome assembly. Comparative genome analysis showed plant metabolic gene cluster-centric evolution of key medicinal metabolites. The chromosome-scale genome assembly of *G. uralensis* offers a valuable resource to facilitate genome-based breeding in licorice and to explore the emergence of specialized metabolites in Fabaceae lineages.

## 2. Materials and methods

### 2.1. Plant material and chromosome observation

The *G. uralensis* strain 308-19 used in this study for whole-genome sequencing was kindly provided by Takeda Garden for Medicinal Plant Conservation, Kyoto, Japan. Plants were maintained at 22°C for 16 h a day and 8 h night photocycle. For chromosome images, we used root tips of *G. uralensis* to prepare mitotic chromosome slides following the method previously described.^26^ Briefly, root tips were pre-treated with 2 mM 8-hydroxyquinoline for 3 h at 24°C and subsequently fixed in 3:1 (v/v) ethanol–acetic acid at 18°C for 5 days and stored in 70% ethanol at 4°C until use. The mitotic chromosome slides were prepared by the enzymatic maceration—squash method described by Wang *et al.* with some modifications.^27^ We used an enzyme solution containing 1% cellulase Onozuka RS (Yakult pharmaceutical, Japan) and 0.5% pectolyase Y-23 (Kyowa chemicals, Japan). The chromosomes were observed by an OLYMPUS BX-53 fluorescence microscope after counterstaining with 5 µg/ml 4,6-diamidino-2-phenylindole (DAPI) in Vectashield (Vector Laboratories, USA). All fluorescence images were captured with a CoolSNAP MYO CCD camera (Photometrics, USA) and processed by MetaVue/MetaMorph version 7.8, Adobe Photoshop CS3 v10.0.1.

### 2.2. DNA sequencing and *de novo* assembly optimization

We extracted DNA from the young leaves using the Genomic-tips kit (Qiagen, Germany) for whole-genome sequencing, following the manufacturer’s instructions. Genomic DNA extracted from the leaves was tested for quality on an electrophoresis gel and subsequently sheared with g-TUBE (Covaris, USA) at 1,600 × *g* for six times. The DNA was subjected to HiFi SMRTbell library construction using the SMRTbell Express Template Prep Kit 2.0 (PacBio, USA) according to the manufacturer’s instructions. The resultant DNA library was further fractionated with BluePippin (Sage Science, USA) to eliminate fragments less than 20 kb in size. The DNA library was sequenced using a single 8M SMRT cell on the Sequel IIe system (PacBio, USA). HiFi sequencing datasets were acquired from the raw data using the SMRT LINK software v11.0 (https://downloads.pacbcloud.com/public/software/installers/smrtlink_11.0.0.146107.zip). To derive *de novo* genome assembly for *G. uralensis*, we first tested Canu v2.2,^28^ Falcon unzip,^29^ and Hifiasm program^30^ using default parameters and subsequently tested different parameters for Hifiasm to optimize and derive primary contig-level genome assembly for *G. uralensis*. Primary assembly was then analysed by purge_dup program^31^ using default parameters to remove haplotigs and polished using Nextpolish software^32^ and HiFi sequencing datasets. The primary assembly thus obtained was used for further scaffolding using Hi-C library sequencing datasets. We used HiFi reads to estimate heterozygosity within *G. uralensis* genome assembly using Jellyfish v2.2.6^33^ with Kmer of 21 and Genomescope2 program,^34^ which suggested genome size as 397 Mb, similar to the genome size estimated previously.^25^

### 2.3. Hi-C library preparation and scaffolding

Following the manufacturer’s instructions, Hi-C libraries were prepared using Arima kit-1 (San Diego, USA). Briefly, 1 g of the young leaf was sectioned using scissors into small pieces (0.5–1 cm) and fixed using 1% formaldehyde (diluted using double autoclaved water) under vacuum while maintaining tissues on the ice for 30 min with mixing samples every 10 min. Tissue fixing was stopped by adding glycine and subsequently washed with water for two cycles before snap freezing in liquid nitrogen, and samples were stored at −80°C. Fixed plant tissues were homogenized, and the Hi-C experiment was performed as instructed by the Arima Hi-C kit-1 protocol (document part number-A160135_v00). DNA samples post-Hi-C experiment were fragmented using Covaris to an average size of 450 bp. Illumina libraries were prepared using the Accel-NGS kit (Integrated DNA Technologies, USA) following the manufacturer’s instructions and sequenced on the Illumina NovaSeq sequencer (Illumina, USA) in the paired-end mode with a read length of 150 bp. Hi-C library sequencing datasets were trimmed to remove adaptors and low-quality bases. For scaffolding, we used 3D-DNA pipeline^35^ under default parameters. Scaffolding was followed by assembly gap-filling using the TGS-Gapcloser program.^36^ Scaffolded and gap-closed assembly was mapped with Hi-C library reads and analysed using Juicer program^37^ to identify any potential errors or misassemblies, and further verified by mapping HiFi reads to the genome assembly using minimap2 program.^38^ Verified *G. uralensis* genome assembly was subsequently used for gene prediction and genome characterization.

### 2.4. Gene prediction, repeat analysis, and annotation

*Glycyrrhiza uralensis* gene prediction was performed as previously described.^39^ Briefly, we used BRAKER2 v2.1.0 program^40^ with RNA-seq datasets mapped to the genome assembly as evidence for gene prediction. The RNA-seq datasets used for expression evidence were obtained previously (Bioproject id: PRJDB2812)^41^ (Supplementary Table S1). RNA-seq datasets were mapped to the genome assembly using Hisat-2 v2.2.0 program,^42^ and expression data were acquired as previously described.^43^ The predicted gene sets were used as queries against the UniProtKB database [https://www.uniprot.org (15 March 2022, date last accessed)] with *E*-value cut-off as 1e−10 and identity 98% using DIAMOND v0.9.29.13038 program^44^ in ‘more-sensitive’ mode. We used the BLASTP program to search homologues in the NCBI-nr database (ftp://ftp.ncbi.nlm.nih.gov/blast/db/FASTA/) with *E*-value 1e−10 and the maximum number of hits as 20. We also performed a BLASTP-based homologue search against the protein sequences of *Arabidopsis thaliana* and *Medicago truncatula* with *E*-value 1e−50 and 90% length coverage. We classified gene sets into three categories, namely, high-confidence (HC) genes, low confidence (LC)/pseudogenes, and transposable elements (TEs). The genes with protein database annotation and TPM values >0 were classified as HC genes, while genes having hits with keywords related to TEs were classified as *de novo* TEs. The remaining genes were regarded as LC/pseudogenes. We used only HC genes for comparative genomics, gene cluster analysis, phylogenetic analysis, and annotations (from hereafter, *G. uralensis* genes). We used InterProScan program^45^ and eggNOG mapper v2.0^46^ to classify *G. uralensis* genes into protein families. The genes were also searched against the Pfam v33.1 database with *E*-value ≤1e−80 by HMMER v3.2.1.^47^ Annotated gene models were functionally mapped, validated, and assigned with gene ontologies using OmicsBox software (Biobam, Spain). *Glycyrrhiza uralensis* genome and predicted gene models were benchmarked using BUSCO v5.3.2.^48^ We mapped unigenes from *G. uralensis* transcriptome assemblies using BLAT software^49^ and *G. uralensis* Illumina sequencing datasets from previously published genome assembly using Bowtie 2.0^50^ to access genome assembly quality.

We used the RepeatModeler v1.0.11 program^51^ (http://www.repeatmasker.org/) to predict repetitive sequences in the *G. uralensis* genome. The repetitive sequences were searched against Repbase^52^ (http://www.girinst.org/repbase/) and were annotated by RepeatMasker v4.0.7,^53^ and the *G. uralensis* genome was hardmasked to use for the PlantClusterFinder program.^54^ Tandem repeats of the *G. uralensis* genome were identified using Tandem Repeats Finder (TRF) program.^55^*Glycyrrhiza uralensis* non-coding RNAs were annotated using multiple software packages and databases. For tRNA identification, we used tRNAscan-SE software^56^ with default parameters. We used INFERNAL v1.1.4 software^57^ against Rfam14 database^58^ to identify and annotate microRNAs, rRNA, and small nuclear RNA (snRNA) coding genes.

### 2.5. Comparative genome analysis

For comparative genome analysis, we used *G. uralensis* gene models with 11 other plant genomes; namely, *A. thaliana*, *Cajanus cajan*, *Cicer arietinum*, *Glycine max*, *Lupinus angustifolius*, *M. truncatula*, *Nelumbo nucifera*, *Ophiorrhiza pumila*, *Vitis vinifera*, *Solanum lycopersicum*, and *Theobroma cacao*, obtained from NCBI genome database. Protein sequences for these 12 plant species were used as input for the OrthoFinder v2.5.4 program^59^ to classify proteins into orthologous and paralogous gene families using the following parameters: using the following parameters: -S blast -t 70 -M msa -A muscle -T raxml-ng -I 1.5. Single copy genes across 12 plant species were aligned using the muscle v5.1 program,^60^ gaps were removed using trimaAl v1.4 program,^61^ and a super-alignment matrix was derived by concatenating individual alignments. The concatenated alignment was subsequently used to derive the species tree using RAxML v8.2.11 program.^62^ The derived species tree was next used for estimation of divergence time using the MCMCtree program from PAML package^63^ implemented in the phylogenetic analysis by maximum likelihood. The estimation of divergence time was performed as previously described. We calibrated the model using divergence time between *M. truncatula* and *C. arietinum* (30–54 MYA), *T. cocoa* and *A. thaliana* (83–93 MYA), and *O. pumila* and *S. lycopersicum* (72–104.9 MYA), obtained from the TimeTree database.^64^ We estimated the gain, expansion, loss, and contraction of orthogenes using orthogene count data for 12 plant species and species phylogenetic tree as input to COUNT software^65^ to perform family history analysis by Wagner parsimony.^66^ Fisher’s Exact test for orthogene families specific to the *G. uralensis* genome when compared with 11 other plant genomes was performed using Omics box software^67^ (Biobam, Spain). We used genes assigned to *G. uralensis*-specific orthogene families as a test set and *G. uralensis* gene models as a reference set and performed a one-tailed Fisher’s Exact test with *P*-value cut-off used as 0.05.

### 2.6. Whole-genome duplication analysis

To explore signs of whole-genome duplication (WGD) in *G. uralensis* genome assembly, we performed synonymous substitution rate (*Ks*) analysis. We identified paralogues for selected plant genomes, including for the *G. uralensis* genome as previously described.^26^ The *Ks* for paralogous gene pairs were estimated using the codeml program^68^ from the PAML package.^63^ We also identified orthogroups between *G. uralensis* and selected plant species using reciprocal BLASTP search and used gene pairs to perform sequence alignment using the MUSCLE program.^60^ The *Ks* value for the reciprocal blast hit pairs was calculated using the codeml program of the PAML package.^63^ To investigate local genome reorganization and WGD, we performed inter- and intra-synteny analysis for *G. uralensis* genome with selected plant species using MCScan program^69^ offered through JCVI package (https://github.com/tanghaibao/jcvi). For synteny analysis, we used genes anchored to eight chromosomes of *G. uralensis*.

### 2.7. Plant metabolic gene cluster analysis

Plant metabolic gene cluster analysis for *G. uralensis* genome assembly was performed as previously described.^26^ Briefly, we used the E2P2 program^70^ to assign protein classification and enzyme identity to *G. uralensis* genes. We next used E2P2-based protein identity and MetaCyc reaction identifiers for pathway inference and *G. uralensis* pathway database construction using Pathologic software (v22.5).^71^ The derived pathway was firstly curated manually and then analysed by SAVI software^54^ to exclude any non-plant-related or redundant pathways and used as input together with hardmasked *G. uralensis* genome assembly and annotation structure for the PlantClusterFinder software^54^ using default parameters. For metabolic gene cluster identification, we excluded scaffolds and associated gene models that were not anchored to any of the chromosomes. To identify metabolic gene clusters associated with glycyrrhizin biosynthesis, we used functionally characterized genes assigned to recently published complete biosynthesis pathways^72^ and performed BLASTP search and reciprocal BLASTP search using *G. uralensis* genome assembly as query. The identified genes were then checked within metabolic gene cluster list identified as described above.

## 3. Result and discussions

### 3.1. Establishing chromosome-scale genome assembly for *Glycyrrhiza uralensis*

**Figure 1.**
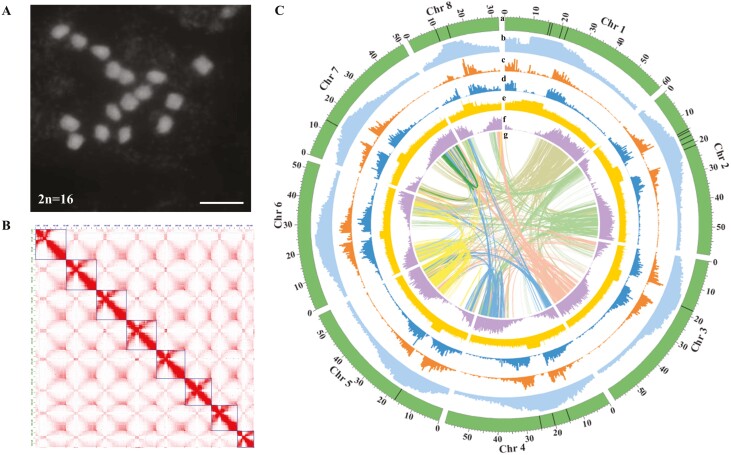
Genome characteristics of *Glycyrrhiza uralensis*. (A) Fluorescence image of DAPI-stained mitotic chromosomes (2*n* = 16). Scale bar = 5 mm. (B) Hi-C contact map for *G. uralensis* genome assembly post scaffolding, polishing, and gapclosing using a stepwise genome assembly approach. (C) Circos plot depicting genomic features. From outer to inner circles: a, chromosomes; b, repetitive sequences; c, distribution of LTR-Gypsy; d, distribution of LTR-Copia; e, %GC density; f, distribution of HC gene models; g, intra-synteny blocks. Assembly gaps have been shown in black colour lines on individual chromosomes (track a). The track a, representing chromosomes, is scaled to the chromosome lengths in Mb.

To achieve a high-quality genome assembly for *G. uralensis*, we selected 308-19 strain (diploid, 2*n* = 16) (Fig. 1A), which was previously assembled using mate-pair short read sequencing datasets.^25^ High molecular weight genomic DNA was sequenced using a single cell of Pacbio Sequel 2 in the HiFi mode, resulting in 15.43 Gb raw PacBio HiFi reads, which corresponds to the 38.9 times the estimated genome size. Heterozygosity analysis using HiFi reads suggested 1.65% heterozygosity with an estimated genome size of 391.9 Mb (Supplementary Fig. S1). With a high heterozygosity level observed in *G. uralensis*, we opted to test different assemblers and performed parameter optimization to derive a highly contiguous genome assembly of *G. uralensis* (Supplementary Fig. S2). Using default parameters, we performed genome assembly using Canu,^28^ Falcon-unzip,^29^ and Hifiasm program.^30^ Primary assemblies obtained using Canu and Falcon-unzip assemblers resulted in genome assembly of nearly double the size of expected genome assembly with contig N50 as 9.78 and 11.73 Mb, respectively (Supplementary Table S2). Compared with these, primary genome assembly using the Hifiasm program with Hi-C libraries under default parameters for phasing resulted in contig N50 as 28.95 Mb with genome size relatively closer to the expected genome size of *G. uralensis*. Therefore, we opted for the Hifiasm program for further parameter optimization. We adjusted parameters to handle high repeat content and heterozygosity of *G. uralensis* genome and achieved the best result for primary assembly with contig N50 as 32.71 Mb and genome size as 449.30 Mb (Supplementary Table S2). Parameter optimization showed relatively lesser influence of varying kmer for Hifiasm program in *G. uralensis* genome assembly, while increasing number of composed reads within a unitig (‘-n’; parameter 6) significantly improve genome assembly (Supplementary Table S2). Increasing minimum coverage cut-off of primary assembly resulted in fragmented genome assembly (parameter 5), most likely due to high heterozygosity and repeat content of *G. uralensis* genome.

We next removed duplicated contigs by processing primary assembly with the purge_dup program,^31^ resulting in the genome assembly in 54 contigs with contig N50 as 36.02 Mb, which we used for further scaffolding (Table 1). We polished primary assembly using HiFi reads and Nextpolish program^32^ and subsequently used it for scaffolding using Hi-C library datasets using the 3D-DNA pipeline (Supplementary Table S2). The resulting scaffolded assembly was next gap-filled using TGSGapclosed^36^ and validated, and error corrected using Juicer software.^37^ Hi-C contact map showed a perfectly scaffolded genome assembly into eight pseudomolecules corresponding to its eight chromosomes, suggesting an accurate genome assembly of *G. uralensis* (Fig. 1B). The finalized genome assembly of *G. uralensis* constitutes 89 scaffolds, with 93.44% of scaffolds anchored to 8 chromosomes with a genome assembly size of 429 Mb and scaffold N50 as 60.2 Mb (Table 1). We detected telomeres at the end of each of the eight chromosomes, suggesting the accuracy of our genome assembly and approach used in this study. The eight chromosomes of *G. uralensis* represented just 17 assembly gaps, with full-length telomere to telomere assembly achieved for chromosome 6 and single gaps for chromosomes 3, 5, and 7 (Fig. 1C and Table 2). With half of the assemblies having single chromosome gap, the *G. uralensis* assembled genome in this study represents the best plant genome within the Fabaceae family and a valuable resource. BUSCO analysis in genome mode and fabales_odb10 as lineage suggested 98.6% of genome completion (Table 1). We further mapped Illumina reads generated for *G. uralensis* in previously published genome assembly to newly assembled genome and observed 98.79% of reads being correctly mapped. We also mapped previously reported RNA-seq datasets for root and leaf tissues of *G. uralensis* and observed a mapping rate within the 94.46–96.63% range (Supplementary Table S3). These results suggest that the genome assembly of *G. uralensis* assembled in this study is of high quality and accurate.

**Table 1.** The assembly statistics of the finalized *Glycyrrhiza uralensis* genome assembly using PacBio HiFi technology and Hi-C library-based scaffolding

Statistics	Contigs	Chromosomes	Unplaced scaffolds
Total number	54	8	81
Total assembly length (bp)	459,188,803	429,071,560	30,142,561
Number of gaps	—	17	3
Average length	8,503,496.35	53,633,945	372,130
Maximum length	59,791,753	60,247,861	4,283,972
Minimum length	35,675	32,253,389	2,000
N50 length	36,026,944	58,560,175	2,547,479
L50 length	5	4	—
GC content (%)		37.13%	47%
BUSCO completeness (genome mode using fabales_odb10 as lineage)	—	98.6%	—

**Table 2.** The annotation statistics of *Glycyrrhiza uralensis* genome assembly across eight chromosomes

	Chr1	Chr2	Chr3	Chr4	Chr5	Chr6	Chr7	Chr8
Size	60,247,861	59,688,110	59,058,686	58,560,175	56,257,782	52,374,569	50,630,988	32,253,389
Number of gaps	4	5	1	3	1	0	1	2
GC %	38.62	37.38	36.7	37.07	36.72	36.25	36.64	37.68
Protein (HC gene)	4,248	4,706	4,733	4,328	4,473	3,754	4,003	2,209
rRNA	13,501	1	2	11	2	0	1	0
tRNA	83	62	81	79	84	63	65	15
Small nucleolar RNA	44	16	51	50	17	21	25	18
miRNA	22	23	38	17	20	24	27	8
Other RNA	30	29	34	49	34	44	25	32
Pseudogene	4,332	2,836	3,373	3,654	1,672	1,930	2,376	1,509

### 3.2. Repetitive contents of *Glycyrrhiza uralensis* genome

Repeat analysis for the *G. uralensis* genome showed that 61.7% (283.1 Mb) of the entire genome constitutes of repetitive elements (Supplementary Table S4). Long terminal repeat (LTR) retrotransposons are the most abundant class of known repeat classes, constituting 22.22% of all repeats, which was fewer than *G. max*.^73^ Within LTR elements, the dominant repeat class was LTR-Copia (54.7% of total LTR elements), which was different from the repeat content profile for other legume plants, including *G. max*, *M. truncatula*, and *C. cajan* genomes, which constitutes LTR-Gypsy as the main dominant LTR element.^73–75^ The distribution and density of LTR-Copia and LTR-Gypsy showed similar density patterns across all eight chromosomes. *Glycyrrhiza uralensis* genome also constituted of 1.33% long interspersed nuclear elements (LINEs), 1% simple repeats, 0.5% Satellite, and 0.1% short interspersed nuclear elements (SINEs) as other known repeat elements (Supplementary Table S4). Within LINEs repeat class, L1/CIN4 was the dominant repeat type. Further, 7.6% of *G. uralensis* genome also constitutes of Class II DNA elements, of which, hobo-Activator family (2.32%) was the dominant repeat class.

It is important to note that the repeat content identified in previously reported draft genome of *G. uralensis* was estimated as 161 Mb, which constituted only 36.48% of the genome assembly.^25^ The huge difference in terms of genomic repeat contents observed between the two genome assemblies for the same plant is due to the different sequencing technologies used. Repeat sequences pose major computational challenges in order achieve read alignments and assembly. Short reads are not able to assemble repeats, and therefore, results in thousands of assembly gaps, while long reads, even though achieve alignments for repeats, error correction for repeats are often ignored to order to direct computational resources towards genome assembly for relatively less repetitive genomic segments and genes. The fact that almost half of *G. uralensis* genome constitutes of repeats, yet we achieved a genome assembly with just 17 assembly gaps suggests the value of adopting Hifi sequencing approach for the plant resequencing projects. Further, the repeats and TEs identified for *G. uralensis* genome is valuable to compare and explore repeat contents of other Leguminosae and Fabaceae species.

### 3.3. Genome annotation and characterization

*De novo* gene prediction using *G. uralensis* genome identified 32,941 HC genes with expression evidence and homology across multiple protein databases, with 32,454 gene models anchored to eight chromosomes (Supplementary Table S7). BUSCO analysis using *G. uralensis* gene models and embryophyte and Fabaceae lineages showed 96.5% and 95.8% genome completeness, respectively. We also identified 26,539 pseudogene models (Table 2 and Supplementary Table S8) and 15,726 *de novo* TEs (Supplementary Table S9) in *G. uralensis* genome assembly. We used HC gene models for all subsequent comparative genome and gene cluster analyses (from hereafter, *G. uralensis* gene models). On average, we identified 4,056 genes across eight chromosomes, with Chr8 consisting of smallest number of gene models (Table 2). *Glycyrrhiza uralensis* gene models were annotated using NCBI-nr databases, Swiss-Prot databases, InterPro, and eggNOG, and annotations were merged and mapped, annotated, and verified using the blast2go from OmicsBox program.

In total, 28,848 gene models (87.57% of gene models) got a homologue across various databases, with 23,165 identified with GO-Slim-based annotation (Supplementary Fig. S3 and Table S7). The top-hit species distribution plot suggested that most top-hit homologues of *G. uralensis* gene models were associated with Fabaceae lineages, with the previously sequenced *G. uralensis* genome being placed at the 23rd position (Supplementary Fig. S3). We observed that in the NCBI-nr database, there are only 184 genes listed, which explained the reason for *G. uralensis* gene models showing such a low percentage of annotation with previously reported genome assembly. BLASTP-based search using *G. uralensis* previously published gene models showed 28,844 gene models being assigned with a homologue. Sequence similarity distribution plot and highest scoring pairs homologues for *G. uralensis* gene models showed a very high score, further validating the annotation pipeline adopted in this study (Supplementary Fig. S3). InterProScan-based annotation assigned different protein domain characteristics to 29,248 *G. uralensis* gene models, which included glycosyltransferases, glycoside hydrolases and cytochrome P450 among some of the top InterProScan protein families (Supplementary Fig. S4). InterProScan IDs distribution plot based on different databases has been shown in Supplementary Fig. S5. Annotation from different databases and programs was used to assign gene ontology (GO) terms to *G. uralensis* gene models using the OmicsBox program (Supplementary Fig. S6). The top GO terms assigned to molecular function category included transferase activity, hydrolase activity, and oxidoreductase activity, some of the active processes that results in the *G. uralensis* characteristic metabolic features. The enzyme code (EC) distribution plot showed transferases, hydrolases, and oxidoreductases among the top three EC classes among *G. uralensis* gene models assigned with enzyme annotation (Supplementary Fig. S7). The known structural diversity of triterpenoid saponins that have been reported in *G. uralensis* is because of enzyme superfamilies, including P450s and UDP-dependent glycosyltransferases (UGTs). GO-based classification and ECs assigned to gene models were consistent with expected gene families that are required to derive metabolic processes in *G. uralensis*.

Annotation for RNA features of *G. uralensis* genome identified 532 tRNAs, 13,518 5S ribosomal RNAs, 179 microRNAs, and 242 small nucleolar RNA among the major categories (Supplementary Tables S5 and S6). The distribution of 5S rRNA was particularly unique to the *G. uralensis* genome as 99.38% of 5S rRNA was identified in Chr1. While several plant genomes have shown the majority of 5S rRNA being located within single or fewer chromosomes, the number of identified 5S rRNA and the majority being assigned to a single chromosome of the *G. uralensis* genome is very interesting.

The peaks for repetitive sequence distribution along chromosomes were perfectly aligned with the assembly gaps (Fig. 1C; track b), representing the putative site of centromeres of *G. uralensis*. We could observe a drop in gene model distribution peak perfectly aligned with the peaks of repetitive sequence distribution along chromosomes (Fig. 1C), which is consistent with the genome architecture of plant species.^26^ It is important to note here that we achieve telomere to telomere assembly for one of the chromosomes, Chr6, suggesting the advantage of PacBio Hifi sequencing technology to achieve a near complete genome assembly even for a heterozygous plant genome.

### 3.4. WGD and expansion of gene families in *G. uralensis* genome

**Figure 2.**
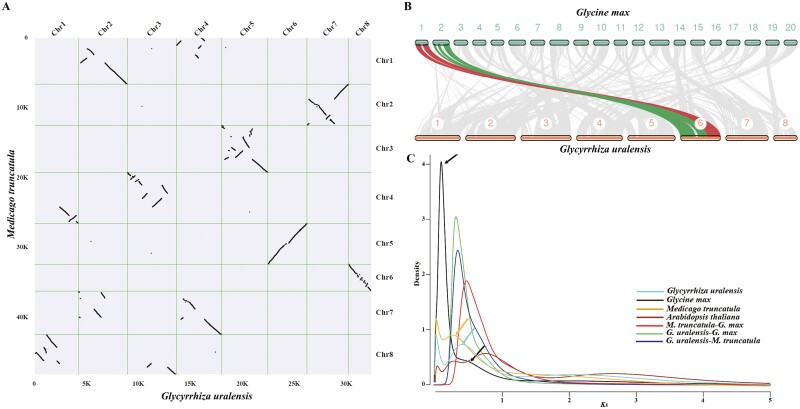
Comparative genomics revealed WGD event in *Glycyrrhiza uralensis* genome. (A) Inter synteny dot plot between genomes of *G. uralensis* and *Medicago truncatula*. A nearly perfect 1:1 synteny relationship was observed for Chr5–8, while rearrangements together with 1:1 synteny was observed for Chr1–4 of *G. uralensis* when compared with *M. truncatula* genome. (B) Inter-synteny chromosome plot between *G. uralensis* and *Glycine max* genome assemblies. Chr1 and Chr2 of *G. max* shows synteny relationships with Chr6 of *G. uralensis*. (C) Synonymous substitution (*Ks*) plot using paralogous and orthologous genes among *G. uralensis*, *M. truncatula*, *G. max*, and *Arabidopsis thaliana* genome assembly.

WGD is among the key evolutionary events that led to the burst of genes with an opportunity towards neofunctionalization, leading to the emergence of specialized metabolites that characterizes a plant species.^23,26,76^ Intra-synteny analysis for *G. uralensis* genome showed 18.32% singleton and 28.35% of gene models as WGD or segmental duplication related, and 2,299 genes as tandem duplicates (Supplementary Fig. S8 and Table S10). Intra-synteny dot plot showed a partial duplication within the *G. uralensis* genome, with associations observed between chromosomes (Supplementary Fig. S8). For instance, we observed partial synteny between Chr1 with Chr6 and, to some extent, Chr7. Similar associations were observed between different pairs across eight chromosomes. Overall, we observed 41.1% of genes having dispersed duplications, suggesting extensive local duplications and rearrangements within *G. uralensis* genome. We next performed synteny analysis for *G. uralensis* genome with two other leguminous plant species, namely, *M. truncatula* and *G. max*. Inter-synteny analysis between *G. uralensis* and *M. truncatula* genome showed a 1:1 synteny depth (Fig. 2A and Supplementary Fig. S9A). We observed a perfect 1:1 synteny between chromosome pairs: Chr5–Chr3, Chr6–Chr5, Chr7–Chr2, and Chr8–Chr6 for *G. uralensis* and *M. truncatula* genomes, respectively (Fig. 2A). However, we could also observe partial synteny contacts going beyond 1:1 synteny for the rest of the other four chromosomes, suggesting genomic rearrangements within *G. uralensis* and *M. truncatula* post divergence (Fig. 2A). For instance, we observed perfect collinearity for Chr1 of *G. uralensis* with Chr8 and partial synteny contacts with Chr4 of the *M. truncatula* genome. Inter-synteny analysis between *G. max* and *G. uralensis* genome showed a synteny depth of 2:1, with each chromosome of *G. uralensis* showed synteny with two chromosomes of *G. max* (Fig. 2B and Supplementary Fig. S9B).

Previous studies have described a single WGD event in *M. truncatula* and two WGD events post eudicot WGT events in *G. max*.^73,75,77,78^ Synteny analysis indicated a possible WGD in *G. uralensis* genome. To further confirm, we perform synonymous substitutions (*Ks*) distribution analysis using paralogues of *G. uralensis* genome, which showed a second peak at *Ks* 0.384 other than a peak at *Ks* value 2, which represents the typical gamma (γ) event corresponding to whole-genome triplication (Fig. 2C). The rate of synonymous substitutions per site per year for *G. uralensis* was slower than that of *M. truncatula*.^75^ Using the conserved eudicot whole-genome triplication as ~154 MYA and *Ks* peak 2, we estimated the substitution rate as 6.49 × 10^−^9 mutations per site per year (r) for *G. uralensis*, which is close to the rate of 6.1 × 10^−^9 suggested by Lynch and Connery for dicots.^79^ Using this substitution rate and *Ks* value for the second peak, we estimated WGD in the *G. uralensis* genome at approximately 59.13 MYA. *Ks* distribution plot using paralogous genes plot also revealed a single peak for *M. truncatula* and two peaks for *G. max* genome, consistent with previously published single WGD and double WGD events within *M. truncatula* and *G. max* genomes, respectively (Fig. 2C).^73,75^ Comparative genome analysis has predicted a shared whole-genome triplication event for ancestral eudicots, followed by extensive rearrangements and gene losses that characterized the present plant genomes.^26,80,81^ Further, duplication pattern and genomics comparisons support an additional WGD event approximately 58 million years ago (MYA) in the papilionoids.^75,78,82^ Estimated WGD in *G. uralensis* suggests that it shares the WGD event with *G. max* and *M. truncatula* and with other papilionoids; afterwards, no recent WGD was observed in *G. uralensis* genome.

**Figure 3.**
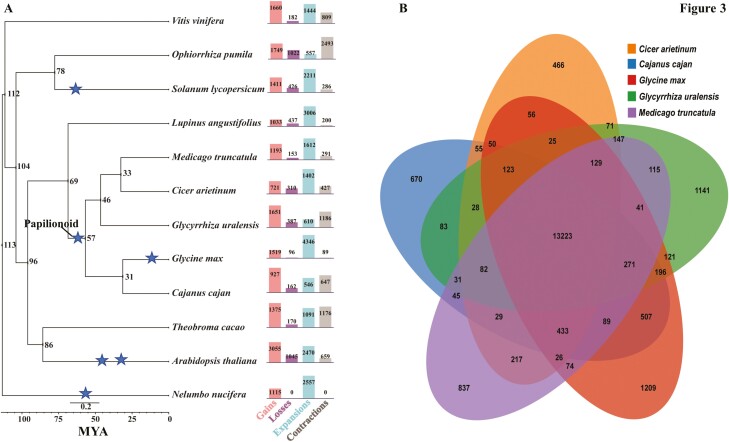
Phylogenetic relationships of *Glycyrrhiza uralensis* genome together with 11 other plant genomes. (A) Phylogenetic tree of 12 plant species using single copy gene families conserved across the selected plant species. The divergence time was estimated using MCMCTree and indicated at the nodes of the phylogenetic tree in MYA. Gene family gain, loss, expansion, and contraction were calculated using Wanger’s parsimony and orthogene family count datasets. The number depicted on the bar chart next to the phylogenetic tree represents number of orthogene families undergone changes. The stars represent WGD events. (B) Venn diagram of shared and unique orthogene families among five legume plants used for phylogenetic analysis.

We next analysed the *G. uralensis* genome with 11 other plant species to estimate divergence time and evolutionary position. Among these 12 plant species, we identified 29,110 orthologous families with 53 single copy families (Supplementary Table S11). Based on the single copy orthogenes, we constructed phylogenetic relationships between 12 plant species and subsequently estimated divergence time using MCMCtree analysis^63^ (Fig. 3A). According to the phylogenetic relationships, at approximately 57 MYA, *G. max* diverged from *G. uralensis*, followed by the divergence of *G. uralensis* from *M. truncatula* at approximately 46 MYA (Fig. 3A). It is proposed that the papilionoids radiated into several clades just after WGD, the largest being split into Hologalegina (*M. truncatula*) and the milletioids (*G. max* and other phaseoloids) subclades at about 54 MYA,^83^ which is also close to what we observed in with this study. Overall, 29,456 gene models of *G. uralensis* were assigned to 15,826 orthogene families, while 3,485 genes remained unassigned (Supplementary Table S11). Within *G. uralensis* assigned orthogene families, 9,172 were represented by a single gene, while 15,471 genes (53.14% of *G. uralensis* genes assigned to an orthogene family) were represented by 6,280 orthogene families with two to five genes members, several of which were identified as tandem repeats (Supplementary Table S11). We also identified 917 *G. uralensis*-specific orthogene families, representing 3,201 gene models of *G. uralensis*. Gene enrichment analysis using *G. uralensis*-specific orthogene families using Fisher’s Exact test identified 11 enzyme families, including mannosyl-oligosaccharide glucosidase, 3-isopropylmalate dehydrogenase, and deoxyhypusine synthase (Supplementary Fig. S10A). Gene ontologies for genes specific to *G. uralensis* showed several biological processes being enriched including meristem development and meristem maintenance along with carotene and terpene catabolic processes (Supplementary Fig. S10B). Gene enrichment analysis showed *G. uralensis*-specific processes that may have shaped its metabolic properties including medicinally relevant metabolites.

Among orthogene families shared across *C. arietinum*, *C. cajan*, *G. max*, *M. truncatula*, and *G. uralensis*, 1,141 orthogene families were specific to *G. uralensis*, while 13,223 (68% of shared orthogenes across these five species) orthogenes were represented by at least one gene from each of these five species (Fig. 3B and Supplementary Table S11). Using Wanger’s parsimony and orthogene family count datasets, we estimated an overall gain in the gene families within the *G. uralensis* genome, which was within a similar range with *G. max* (Fig. 3A). On the other hand, *G. max* showed a significantly higher number of gene families undergoing expansion when compared with *G. uralensis*. *Glycyrrhiza uralensis* genome also showed a relatively higher number of gene family contractions comparable only with *T. cacao* and *O. pumila*, suggestive of an active ongoing purifying process towards its established chemodiversity. Overall, our results identified a shared WGD event across papilionoids in *G. uralensis* genome, with more than half of the genes identified as orthologous (two to five-membered orthogene families), supporting the role of the neofunctionalization hypothesis for specialized metabolite biosynthesis driven by locally duplicated or rearranged genes.^75,84^

### 3.5. Plant gene cluster-centric biosynthesis of glycyrrhizin

Plant metabolic gene clusters have become a contentious topic, where their existence and relevance in identifying new enzymes involved in the biosynthesis of specialized metabolites are supported and questioned simultaneously. Rai *et al.* suggested metabolic gene clusters as the site of preserving metabolic characteristics of the plant by retaining core genes that derive biosynthesis of key metabolic steps.^26^ Several studies have shown the biosynthesis of specialized metabolites centred around the gene clusters across diverse plant species.^26,85–87^ We used contiguous genome assembly of *G. uralensis* to investigate if the biosynthesis of glycyrrhizin, the major saponin synthesized in the licorice, is also centred around metabolic gene clusters. E2P2 software-based enzyme classification^70^ was used to derive MetaCyc reaction identifiers.^88^ Subsequently, as previously described,^26^ the *G. uralensis* metabolic pathway database was constructed through manual inspection, SAVI software-driven automated inspection, and PathoLogic software.^71^ In total, 499 metabolic pathways were assigned to 5,929 peptides with 2,728 enzymatic reactions and 81 transport reactions. The pathway database for *G. uralensis* has been provided as open-source database, which can be accessed using GitHub repository associated with this study (https://github.com/amit4mchiba/Glycyrrhiza-uralensis-strain-308-19-genome). Using the pathway database and genomic locations, we identified 355 secondary metabolic gene clusters consisting of 3,489 *G. uralensis* genes (Supplementary Tables S12 and S13). Biosynthesis pathways for isoflavonoids biosynthesis I and II using *G. uralensis* pathway database has been shown in Supplementary Figs S11 and S12, with genes assigned to metabolic gene clusters are represented next to the annotated genes.

**Figure 4.**
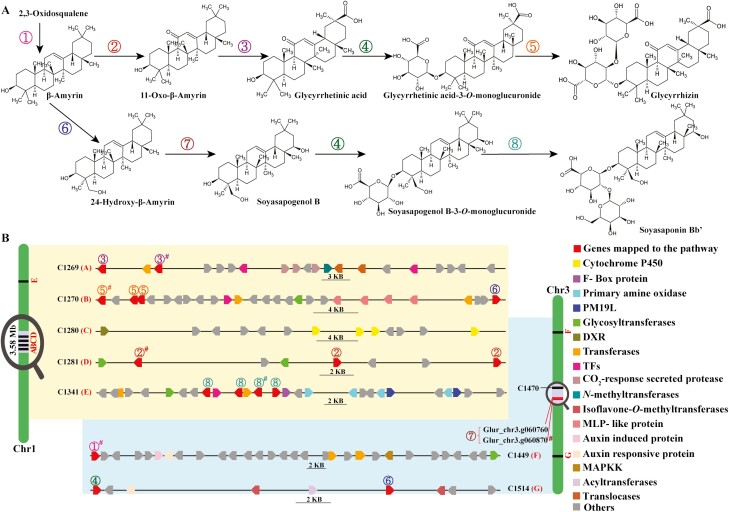
Genes associated with glycyrrhizin biosynthesis are clustered within *Glycyrrhiza uralensis* genome. (A) Biosynthesis pathways for triterpenoid saponins biosynthesis in *Glycyrrhiza uralensis*. (B) Genes assigned to enzymes involved in the biosynthesis triterpenoid saponins and member of gene clusters in Chr1 and Chr3 of *G. uralensis*. Functionally characterized genes were used as BLASTP database, and homologues were identified from *G. uralensis* gene models. Genes marked by ‘#’ are reciprocal blast hits for functionally characterized enzymes.①: β amyrine synthase; ②: CYP88D6; ③: CYP72A154; ④: cellulose synthase-derived glycosyltransferase (CSyGT); ⑤: UGT73P12; ⑥: CYP93E3; ⑦: CYP72A566; ⑧: UGT73P13. DXR, 1-deoxy-d-xylulose 5-phosphate reductoisomerase; MAPKK, mitogen-activated protein kinase kinases; MLP, major latex protein; PM19L, plasma membrane 19-like protein; TFs, transcription factors. The position of genes within gene clusters is scaled.

Within the identified metabolic gene clusters, 125 gene clusters included glycosyltransferase as the signature tailoring enzyme class, while 32 and 67 gene clusters included cytochrome P450 and acyltransferase as the signature tailoring enzyme class, respectively (Supplementary Table S12). Further, 80% of identified metabolic gene clusters (281 out of 355 metabolic gene clusters) showed local duplication of genes. Local duplication has often been described to expand the scope of acquiring a novel enzyme function from an existing one through neofunctionalization, thus deriving plant chemodiversity.^84^*Glycyrrhiza uralensis* gene clusters showed local duplication as a characteristic feature. The propensity of local duplication was similar as observed in *M. truncatula*, which showed a significantly higher percentage of local duplication of genes when compared with *G. max*.^75^ We used functionally characterized genes from the oleanane-type triterpenoid saponins^72^ and used as a database to identify homologues within the *G. uralensis* genome. Using BLASTP and reciprocal BLASTP search, we identified 81 genes corresponding to 10 different enzymes from the glycyrrhizin and/or soyasaponin biosynthesis pathway (Supplementary Tables S14 and S15). Entire biosynthesis pathways for glycyrrhizin biosynthesis were identified as part of partially fragmented gene clusters. Three out of five enzymes involved in the biosynthesis of glycyrrhizin, namely, CYP88D6, CYP72A154, and UGT73P12, were identified on Chr1 as members of gene clusters C1281, C1269, and C1270, respectively (Fig. 4 and Supplementary Fig. S13). The expression of genes, using previously reported RNA-sequencing datasets^41^ (Supplementary Table S1), assigned to gene clusters associated with glycyrrhizin and/or soyasaponin biosynthesis pathways are shown in Supplementary Fig. S13. Glur_chr1_g064610.1 gene, annotated as CYP93E3, which catalyses the conversion of β-amyrin to 24-hydroxy-β-amyrin in soyasaponin biosynthetic pathway, was identified as a member of C1270 gene cluster on the Chr1. The entire length of the genomic fragment including metabolic gene cluster C1269–C1281 on Chr1 is 3.5 Mb, which includes multiple gene clusters represented by glycosyltransferase, cytochrome P450, and transcription factors, including ethylene-responsive transcription factors. The only enzyme, GuCSyGT, which catalyses the conversion of glycyrrhetinic acid to glycyrrhetinic acid-3-*O*-monoglucuronide was not identified on Chr1, although homologues were assigned to different metabolic gene clusters while functionally characterized gene was identified as member of C1644 gene cluster (Supplementary Fig. S14). It is interesting to find GuCSyGT and CYP93E3 as member of C1514 gene cluster for soyasaponin biosynthesis, since GuCSyGT also transfers a glucuronic acid to C3 position of soyasapogenol B, corresponding sapogenin of soyasaponin Bb. Triterpenoid saponin biosynthesis involves formation of various triterpene scaffolds through cyclization of 2,3-oxidosqualene by oxidosqualene cyclases, which subsequently undergoes site-specific oxidation catalysed by cytochrome P450 to deliver diverse triterpenoid aglycones^89,90^ (Fig. 4A). These triterpenoid aglycones are further catalysed by glycosyltransferases 1 superfamily or cellulose synthase-derived glycosyltransferases (CSyGTs) belonging to glycosyltransferase 2 superfamily, resulting in the known saponin diversity in plants.^89–92^ Our results showed a metabolic gene cluster-centric genome architecture for glycyrrhizin and soyasaponin biosynthesis in the *G. uralensis* genome. We also observed biosynthesis of isoflavonoids being associated with metabolic gene clusters (Supplementary Figs S11 and S12). Previously, Mochida *et al.* reported a synteny block among *C. arietinum*, *M. truncatula*, and *G. uralensis* draft genome, which included functionally characterized enzymes involved in the isoflavonoid biosynthesis.^25^ We identified these enzymes being assigned to the gene cluster C1514 on Chr3 (Supplementary Fig. S11).

The gene clusters are the genomic regions with enzyme coding genes associated with catalysing biochemical reactions resulting in metabolite diversity driven by signature and tailoring enzymes. The member genes within a gene clusters, therefore, becomes candidate genes with potential function towards specialized metabolites biosynthesis. While the purpose of gene clusters within the plant genome is still a topic of discussion, there is no denying that it is prevalent across plant genomes. Combing gene clusters with comparative genomics, phylogenomics, and expression analysis could identify candidate genes with relevant functions in the biosynthesis of target metabolites.^23,26,76^ Although gene clusters identified for *G. uralensis* genome are putative and include several uncharacterized enzymes, these offer a resource for researchers to prioritize candidate genes for further characterization and function elucidation. The high-quality genome assembly of *G. uralensis* and metabolic gene clusters identified in this study is a valuable source for exploring the biosynthesis of specialized metabolites and genome-driven species improvement of this valuable medicinal legume plant.

## 4. Conclusions

In this study, we used high-fidelity PacBio sequencing technology to derive one of the most contiguous plant genomes from the Fabaceae family. We achieved a chromosome-scale genome assembly with only 17 assembly gaps and contig N50 as 36.02 Mb, an improvement of over 360% in terms of assembly contiguity compared with the previously reported genome assembly of *G. uralensis*. We showed the importance of parameter optimization to achieve a highly contiguous genome assembly even for highly heterozygous plant genomes. Using *G. uralensis* genome assembly, we identified a shared recent WGD that occurred at approximately 59.02 MYA and used the genome to identify 355 metabolic gene clusters. Metabolic gene cluster analysis identified a prevalent local duplication characteristic that contributes to the present metabolic features of *G. uralensis*. The genomic resource presented in this study addresses the urgent need for a high-quality genome assembly of medicinal legume plants to explore the biosynthesis of specialized metabolites and for genome-guided species improvement.

## Supplementary data

Supplementary data are available at *DNARES* online.

Supplementary Figure S1. *K-mer* distribution plot and estimation of genome heterozygosity using HiFi PacBio datasets using GenomeScope2.0. Using Jellyfish program and *K-mer* of 21 was used to obtain *K-mer* distribution plot, which showed two peaks, representing heterozygous and homozygous peaks in *Glycyrrhiza uralensis* genome.

Supplementary Figure S2. *De novo* genome assembly pipeline used to derive chromosome-scale genome assembly of *Glycyrrhiza uralensis*. We used Hifi Pacbio sequencing datasets and Hi-C library sequencing datasets and performed parameter optimization to achieve a highly contiguous genome assembly.

Supplementary Figure S3. Characteristics of annotated gene models of *Glycyrrhiza uralensis*. (A) Gene model annotation summary. (B) Top-hit species distribution plot. Species for top-hit homologues from protein databases for *G. uralensis* gene models were plotted here. (C) Gene models length distribution plot. (D) Sequence similarity distribution plot. Sequence similarity score of *G. uralensis* gene models against its closest homologue were plotted. (E) Highest scoring pairs (HSPs) distribution over the *G. uralensis* gene model sequences. (F) HSPs distribution over the corresponding hits that were used to annotate gene models of *G. uralensis*. Multiple protein databases and softwares were used to annotate *G. uralensis* gene models and assigned with functional classification using OmicsBox program. Here, we used gene models labelled as high-confidence gene models, 32,941 in total.

Supplementary Figure S4. InterProScan-based annotation and protein domain characterization of *G. uralensis* gene models. *Glycyrrhiza uralensis* gene models were used as query and InterProScan-based protein domain annotation and classification was performed using multiple source databases, with InterProScan sites (A), family classifications (B), protein repeat classification (C), and protein domain (D) distribution plots have been shown here. The protein classification datasets were used together with homologue-based annotation to assign gene ontologies to *G. uralensis* gene models.

Supplementary Figure S5. InterProScan IDs distribution plot for *G. uralensis* gene models. Annotations based on InterProScan IDs from (A) FPrintScan database, (B) High-quality Automated and Manual Annotation of Proteins (HAMAP) database, (C) SuperFamily database, and (D) Pfam database. InterProScan IDs were merged with annotations using eggNOGs and used to assign gene ontology and enzyme IDs to *G. uralensis* gene models.

Supplementary Figure S6. Gene ontology (GO) distribution plot for *Glycyrrhiza uralensis* genome assembly. Annotations using multiple databases and protein domain classification tools were used to annotate and validate using OmicsBox program, and subsequently used to assign GO terms into three major categories, namely, biological process, molecular function, and cellular component.

Supplementary Figure S7. Annotation characteristics based on assigned enzyme codes to the gene models of *Glycyrrhiza uralensis*. Enzyme codes to the annotated *G. uralensis* gene models were assigned based on merged annotations from multiple databases using OmicsBox program. (A) Enzyme code distribution plot. (B) Oxidoreductases annotated enzyme code distribution plot. (C) Transferases annotated ­enzyme code distribution plot. (D) Hydrolases annotated enzyme code distribution plot.

Supplementary Figure S8. Intra-synteny dot plot for *Glycyrrhiza uralensis* genome. 32,454 genes anchored to eight chromosomes were used for synteny analysis using MCScanX program. We observed hints of local/segmental duplication within *G. uralensis* genome assembly.

Supplementary Figure S9. Inter-synteny analysis for *Glycyrrhiza uralensis* genome with *Medicago truncatula* and *Glycine max*. Synteny analysis was performed for *G. uralensis* with two other legume plants and synteny depth was plotted for (A) *G. uralensis* and *M. truncatula*, and (B) *G. max* and *G. uralensis* genomes.

Supplementary Figure S10. Gene enrichment analysis using Fisher’s Exact test for orthogenes specific to *Glycyrrhiza uralensis*. Nine hundred and seventeen orthogene families were identified to be specific to *G. uralensis* when compared with 11 other plant species, representing 3,201 gene models. Specific gene models were used as test sets, *G. uralensis* gene models were used as reference set, and a one-tailed Fisher’s Exact test was performed with *P*-value cut-off used as 0.05. Enriched enzyme names (A) and top 30 gene ontologies identified within specific gene sets (B) have been represented here.

Supplementary Figure S11. Isoflavonoid biosynthesis I pathway from *Glycyrrhiza uralensis* pathway database established in this study. E2P2-based enzyme classification was used to retrieve MetaCyc-based reactions identifiers, and subsequently used as input for PathoLogic tools to establish *G. uralensis* pathway database. Isoflavonoid biosynthesis I was drawn based on MetaCyc (https://metacyc.org/) template, and gene cluster IDs assigned to a given gene ID, has been shown here.

Supplementary Figure S12. Isoflavonoid biosynthesis II pathway from *Glycyrrhiza uralensis* pathway database established in this study. E2P2-based enzyme classification was used to retrieve MetaCyc-based reactions identifiers, and subsequently used as input for PathoLogic tools to establish *G. uralensis* pathway database. Isoflavonoid biosynthesis II was drawn based on MetaCyc (https://metacyc.org/) template, and gene cluster IDs assigned to a given gene ID, has been shown here.

Supplementary Figure S13. Metabolic gene clusters associated with oleanane-type triterpenoid saponins biosynthesis pathways. Genes assigned to enzymes involved in the biosynthesis of saponins in *G. uralensis*. Functionally characterized genes were used as BLASTP database, and homologues were identified from *G. uralensis* gene models. Genes marked by ‘#’ are reciprocal blast hits for functionally characterized enzymes. Gene clusters associated with genes are represented next to the gene IDs. The heatmap represents RNA-seq-based expression for individual genes using previously described datasets reported by Ramilowski *et al.*^41^ Lib1: RNA-seq datasets extracted from roots of 308-19 (high glycyrrhizin-producing) strain in June; Lib-2: RNA-seq datasets from roots of 308-19 strain in December; Lib-3: RNA-seq datasets from roots of 87-458 (low glycyrrhizin-producing) strain in June; Lib-4: RNA-seq datasets from leaves of 308-19 strain in June. The NCBI SRA accessions for the expression datasets are provided in Supplementary Table S1.

Supplementary Figure S14. Genes associated with C1644 gene cluster representing functionally characterized cellulose synthase-derived glycosyltransferase (CSyGT) in *Glycyrrhiza uralensis* genome. Metabolic gene clusters were identified using PlantClusterFinder program. CSyGT coding gene, Glur_chr5.g000300.1 ④, was identified as member of the gene cluster C1644 together with enzymes associated with diverse metabolic processes.

Supplementary Table S1. The RNA-seq public datasets^41^ used for *Glycyrrhiza uralensis* used for annotation and expression analysis.

Supplementary Table S2. Assembler and parameter optimization to achieve primary contig-level genome assembly for *Glycyrrhiza uralensis*.

Supplementary Table S3. RNA-seq datasets^41^ mapping statistics to *Glycyrrhiza uralensis* genome assembly.

Supplementary Table S4. The repeat classification for *Glycyrrhiza uralensis* genome assembly.

Supplementary Table S5. Non-coding RNA identified in *Glycyrrhiza uralensis* genome using Rfam 14 database.

Supplementary Table S6. Annotation of tRNA identified in the *Glycyrrhiza uralensis* genome.

Supplementary Table S7. *Glycyrrhiza uralensis* high-confidence (HC) gene model annotation and functional classifications using multiple databases.

Supplementary Table S8. *Glycyrrhiza uralensis* low confidence (LC)/pseudogene model annotation and functional classifications using multiple databases.

Supplementary Table S9. *Glycyrrhiza uralensis* transposable element (TE) annotation and functional classifications using multiple databases.

Supplementary Table S10. Intra-synteny analysis for *Glycyrrhiza uralensis* genome using MCSCANX.

Supplementary Table S11. Orthogene family classification for *Glycyrrhiza uralensis* gene models with 11 other plant species.

Supplementary Table S12. *Glycyrrhiza uralensis* gene models’ classifications used to derive metabolic gene clusters.

Supplementary Table S13. Metabolic gene clusters assigned to *Glycyrrhiza uralensis* genome assembly.

Supplementary Table S14. Reciprocal blast hit for *Glycyrrhiza uralensis* gene models and functionally characterized genes involved in the oleanane-type triterpenoid saponins.

Supplementary Table S15. BLASTP-based annotation of *Glycyrrhiza uralensis* genome using functionally characterized genes involved in the oleanane-type triterpenoid saponins as BLASTP database.

dsac043_suppl_Supplementary_Figure_S1

dsac043_suppl_Supplementary_Figure_S2

dsac043_suppl_Supplementary_Figure_S3

dsac043_suppl_Supplementary_Figure_S4

dsac043_suppl_Supplementary_Figure_S5

dsac043_suppl_Supplementary_Figure_S6

dsac043_suppl_Supplementary_Figure_S7

dsac043_suppl_Supplementary_Figure_S8

dsac043_suppl_Supplementary_Figure_S9

dsac043_suppl_Supplementary_Figure_S10

dsac043_suppl_Supplementary_Figure_S11

dsac043_suppl_Supplementary_Figure_S12

dsac043_suppl_Supplementary_Figure_S13

dsac043_suppl_Supplementary_Figure_S14

dsac043_suppl_Supplementary_Table_S1

dsac043_suppl_Supplementary_Table_S2

dsac043_suppl_Supplementary_Table_S3

dsac043_suppl_Supplementary_Table_S4

dsac043_suppl_Supplementary_Table_S5

dsac043_suppl_Supplementary_Table_S6

dsac043_suppl_Supplementary_Table_S7

dsac043_suppl_Supplementary_Table_S8

dsac043_suppl_Supplementary_Table_S9

dsac043_suppl_Supplementary_Table_S10

dsac043_suppl_Supplementary_Table_S11

dsac043_suppl_Supplementary_Table_S12

dsac043_suppl_Supplementary_Table_S13

dsac043_suppl_Supplementary_Table_S14

dsac043_suppl_Supplementary_Table_S15

## Data Availability

All sequencing datasets generated in this study have been deposited in the DDBJ database (Experiment: DRX386007–DRX386008; Run: DRR400303–DRR400304) under the BioProject id PRJDB14223, submission id DRA014720, BioSample: SAMD00521547. The genome sequence of *G. uralensis* strain 308-19 has been public under the accession ids BRZY01000001–BRZY01000089. All datasets generated and discussed in this study are available as a supplementary dataset in this manuscript. The pathway database for *G. uralensis*, used for metabolic gene cluster analysis and biochemical pathway visualization, can be downloaded from our GitHub repository—https://github.com/amit4mchiba/Glycyrrhiza-uralensis-strain-308-19-genome. All scripts, supplementary datasets, and intermediate files generated from this study have been deposited to our GitHub repository (link mentioned above).
