# Supplementary figures and images for: Chromosome-scale genome assembly of Glycyrrhiza uralensis revealed metabolic gene cluster centred specialized metabolites biosynthesis
Source: DNA Res. 2022 Dec 20;29(6):dsac043. doi: 10.1093/dnares/dsac043 (PMC9763095; doi:10.1093/dnares/dsac043)

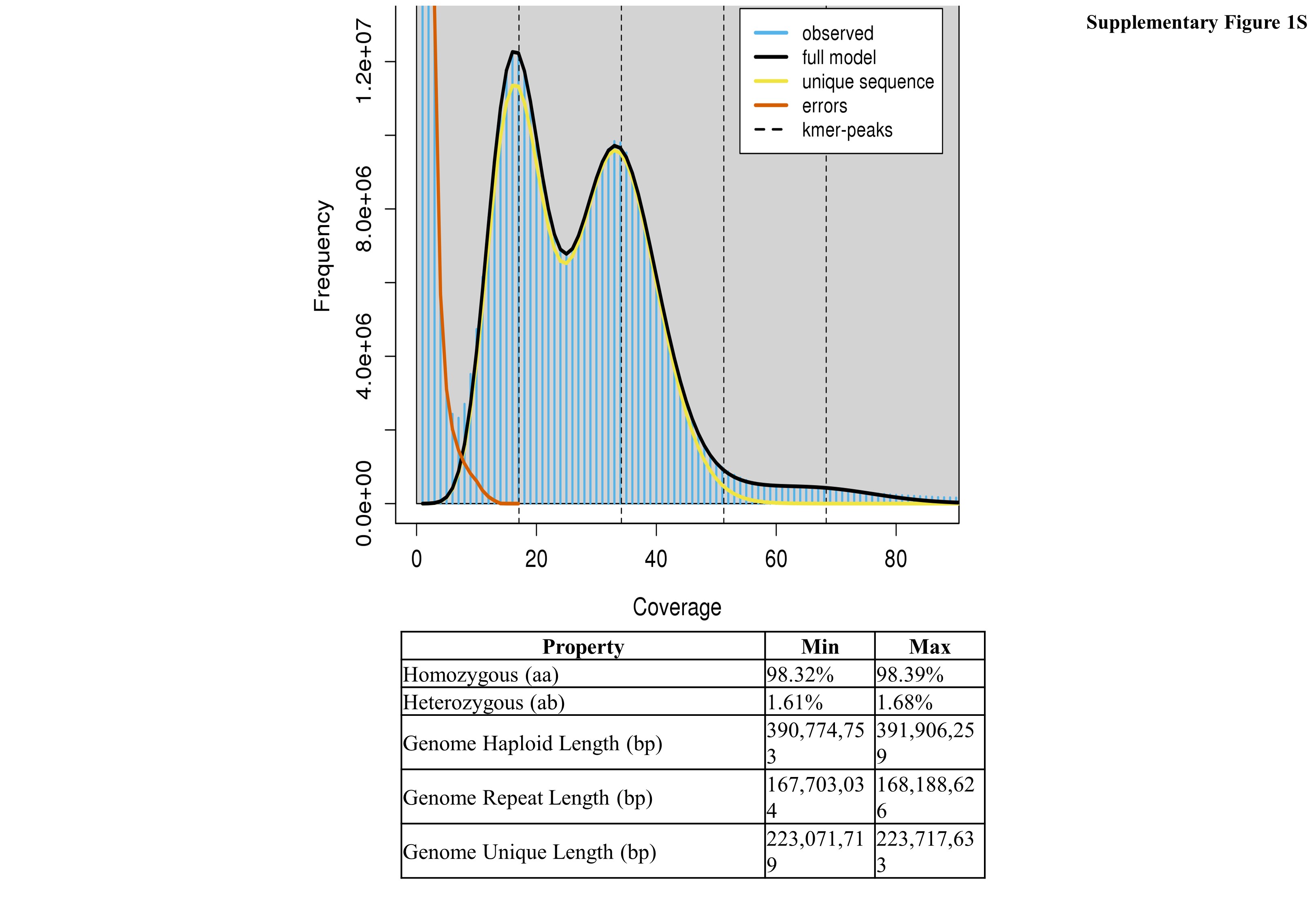

Supplement: dsac043_suppl_Supplementary_Figure_S1 [file dsac043_suppl_supplementary_figure_s1.jpeg]

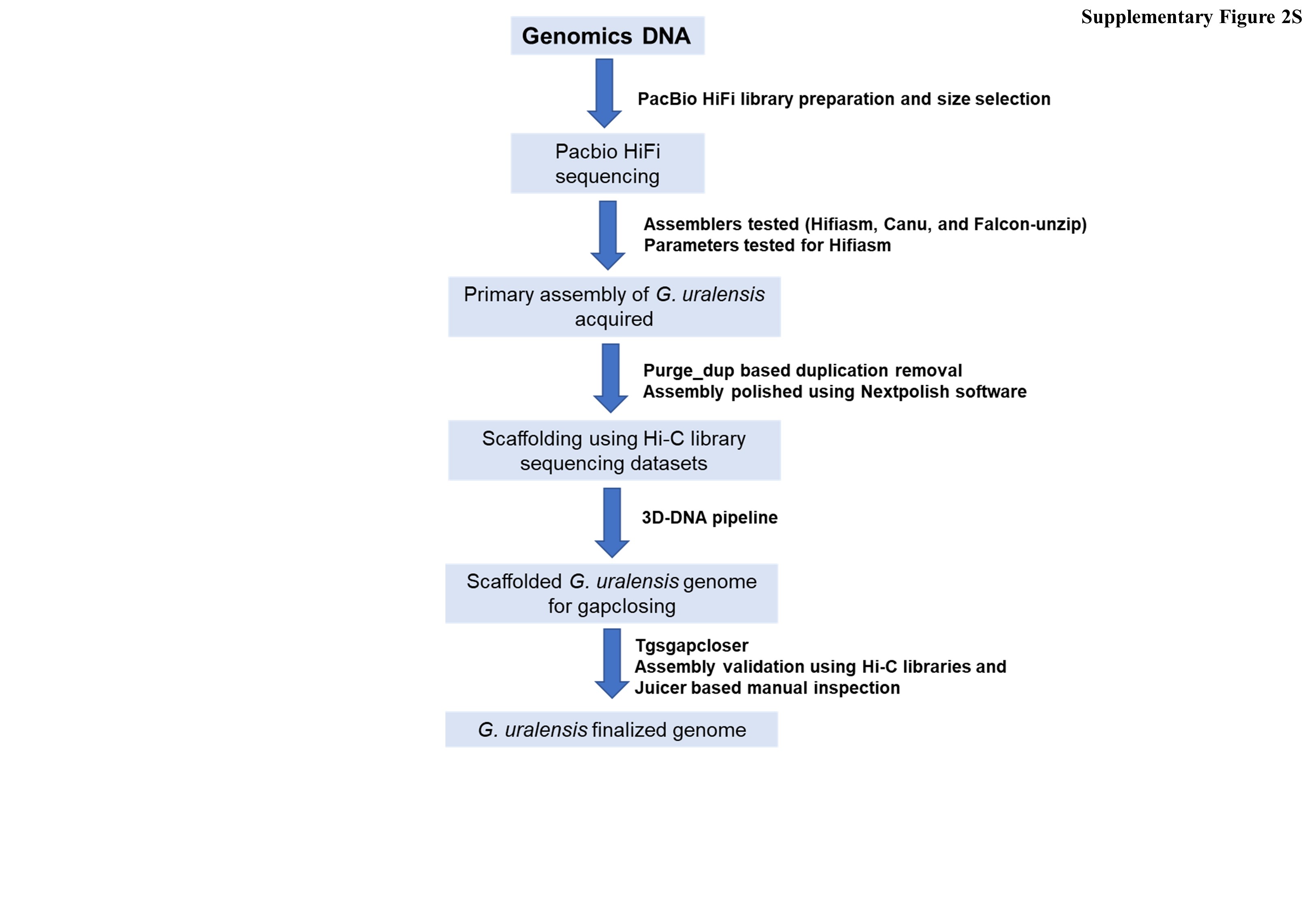

Supplement: dsac043_suppl_Supplementary_Figure_S2 [file dsac043_suppl_supplementary_figure_s2.jpeg]

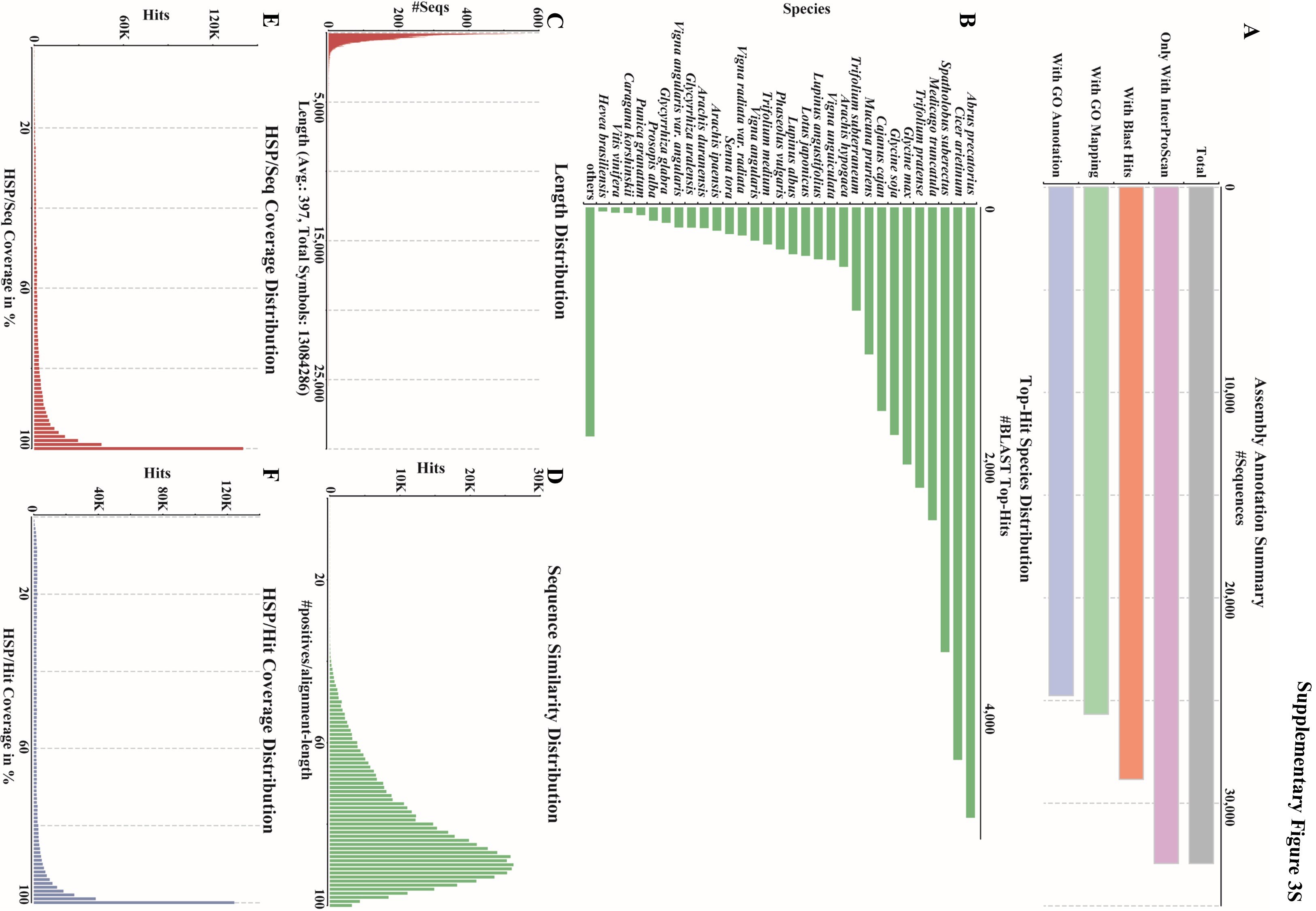

Supplement: dsac043_suppl_Supplementary_Figure_S3 [file dsac043_suppl_supplementary_figure_s3.jpeg]

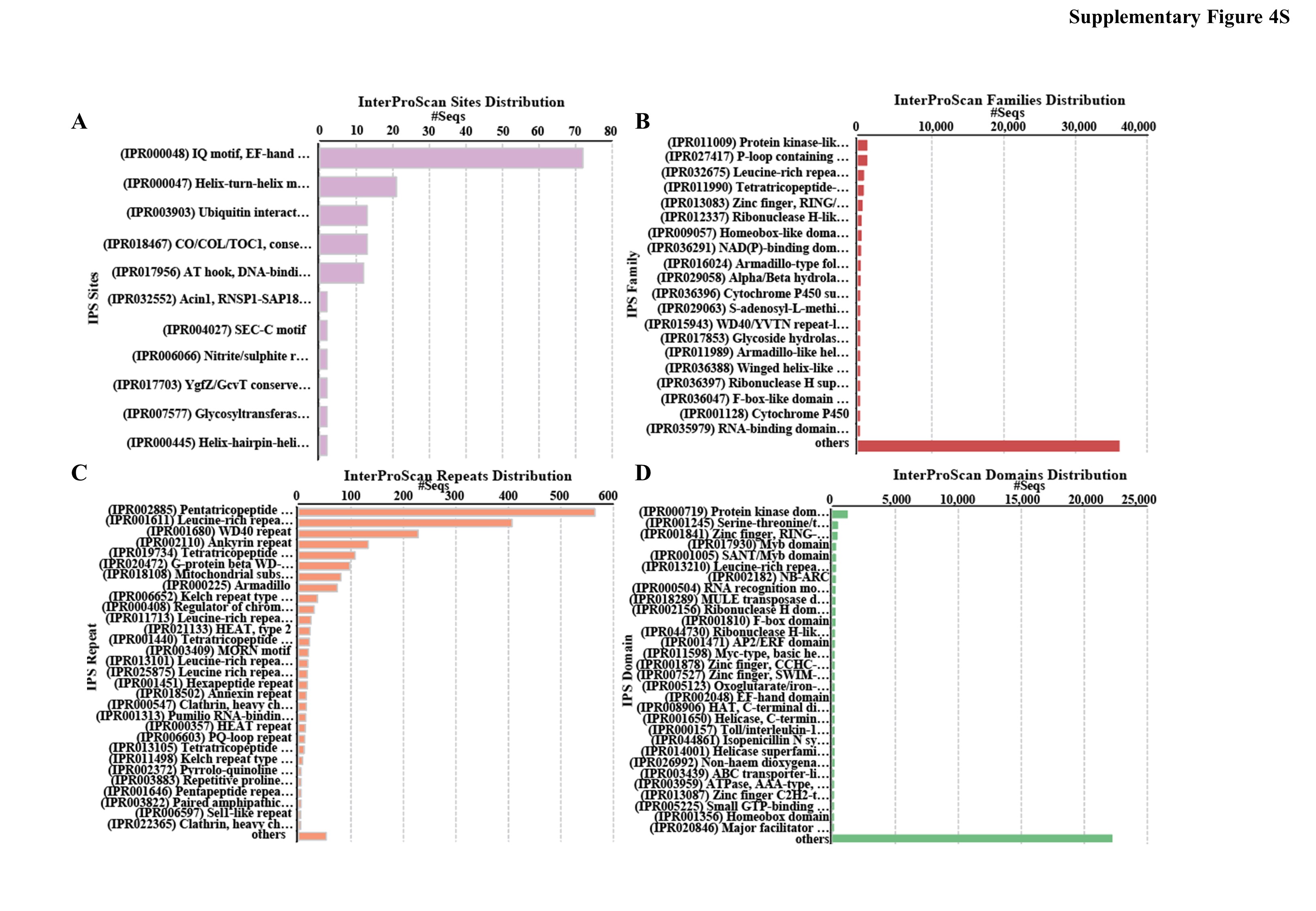

Supplement: dsac043_suppl_Supplementary_Figure_S4 [file dsac043_suppl_supplementary_figure_s4.jpeg]

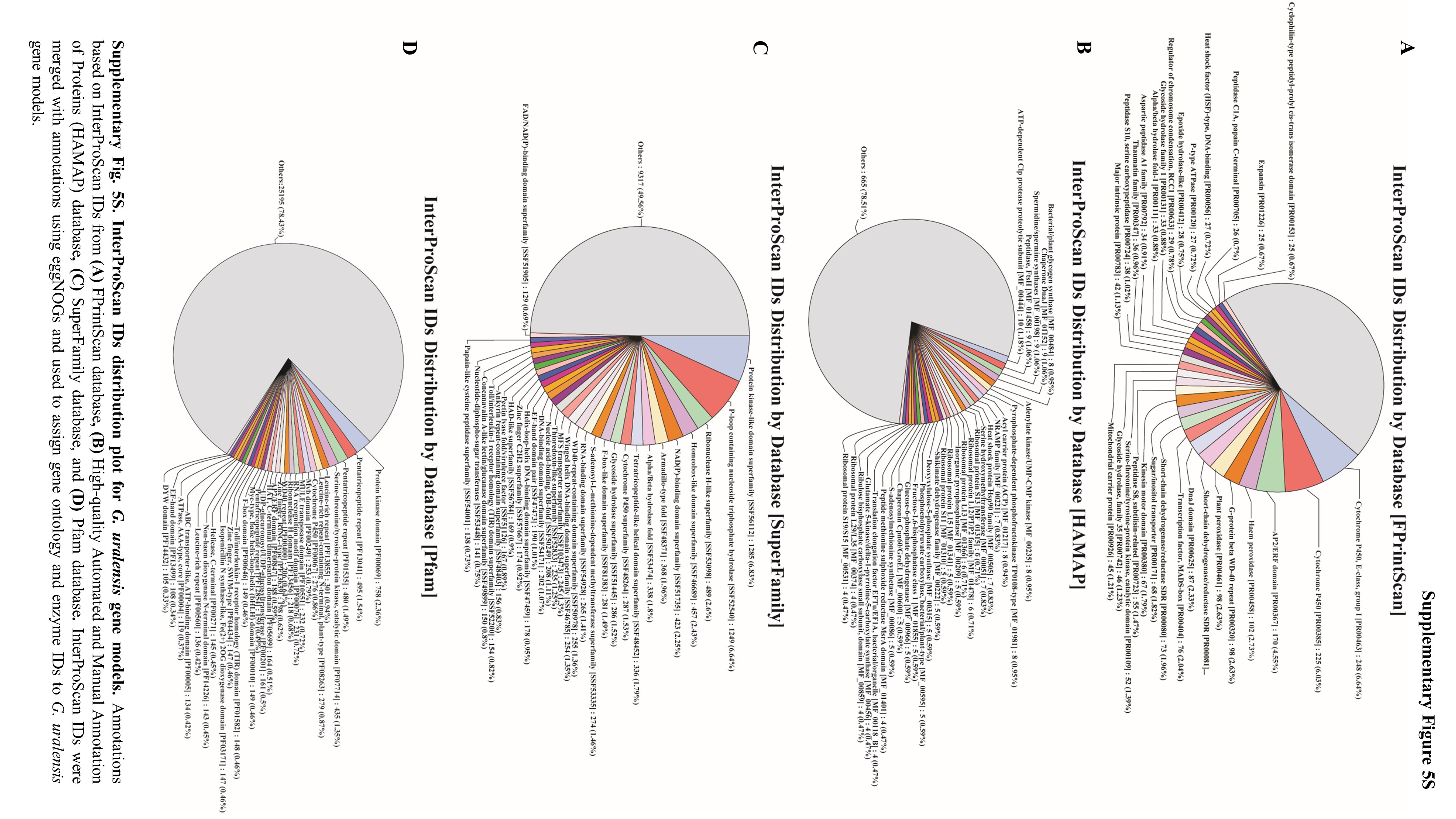

Supplement: dsac043_suppl_Supplementary_Figure_S5 [file dsac043_suppl_supplementary_figure_s5.jpeg]

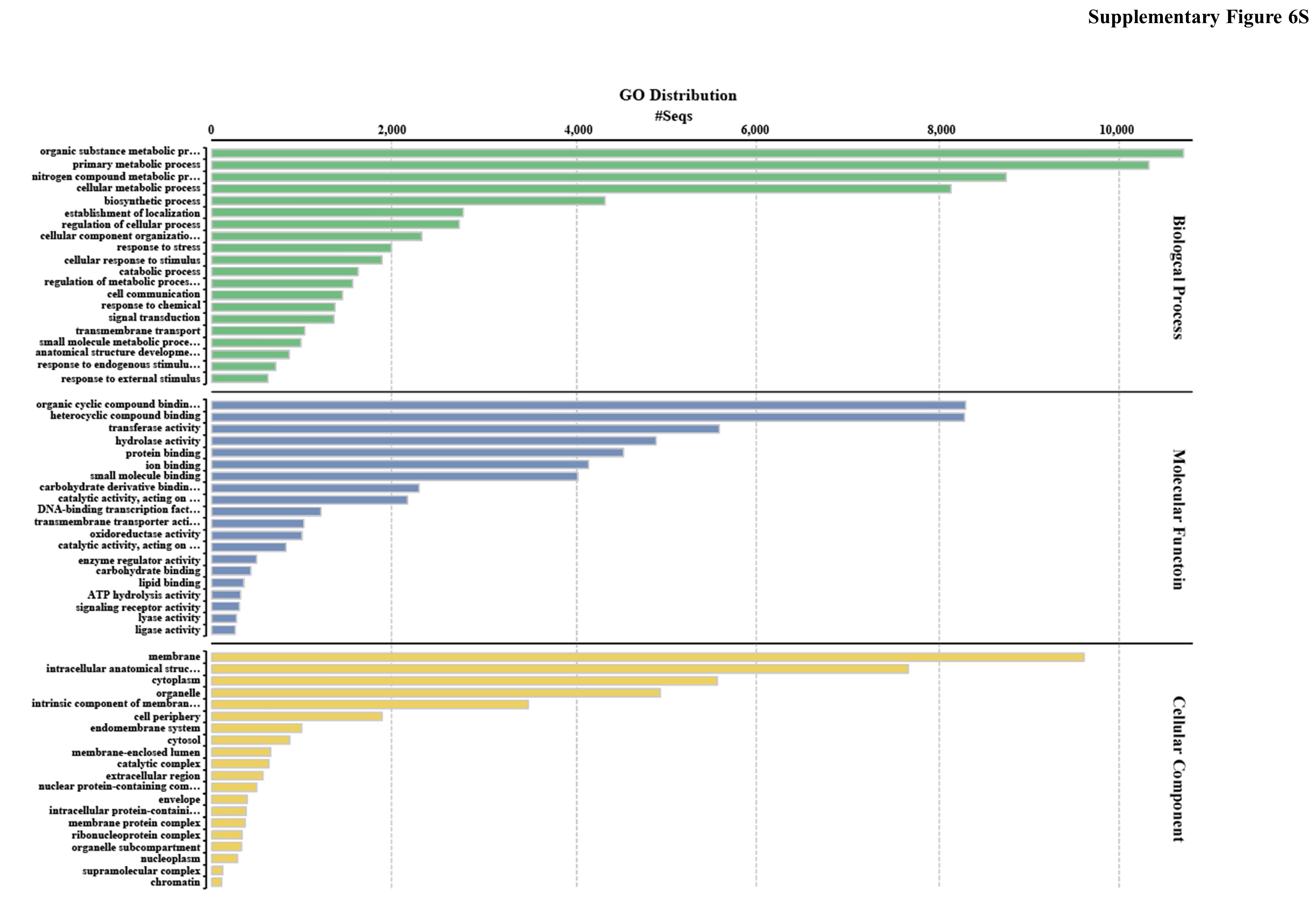

Supplement: dsac043_suppl_Supplementary_Figure_S6 [file dsac043_suppl_supplementary_figure_s6.jpeg]

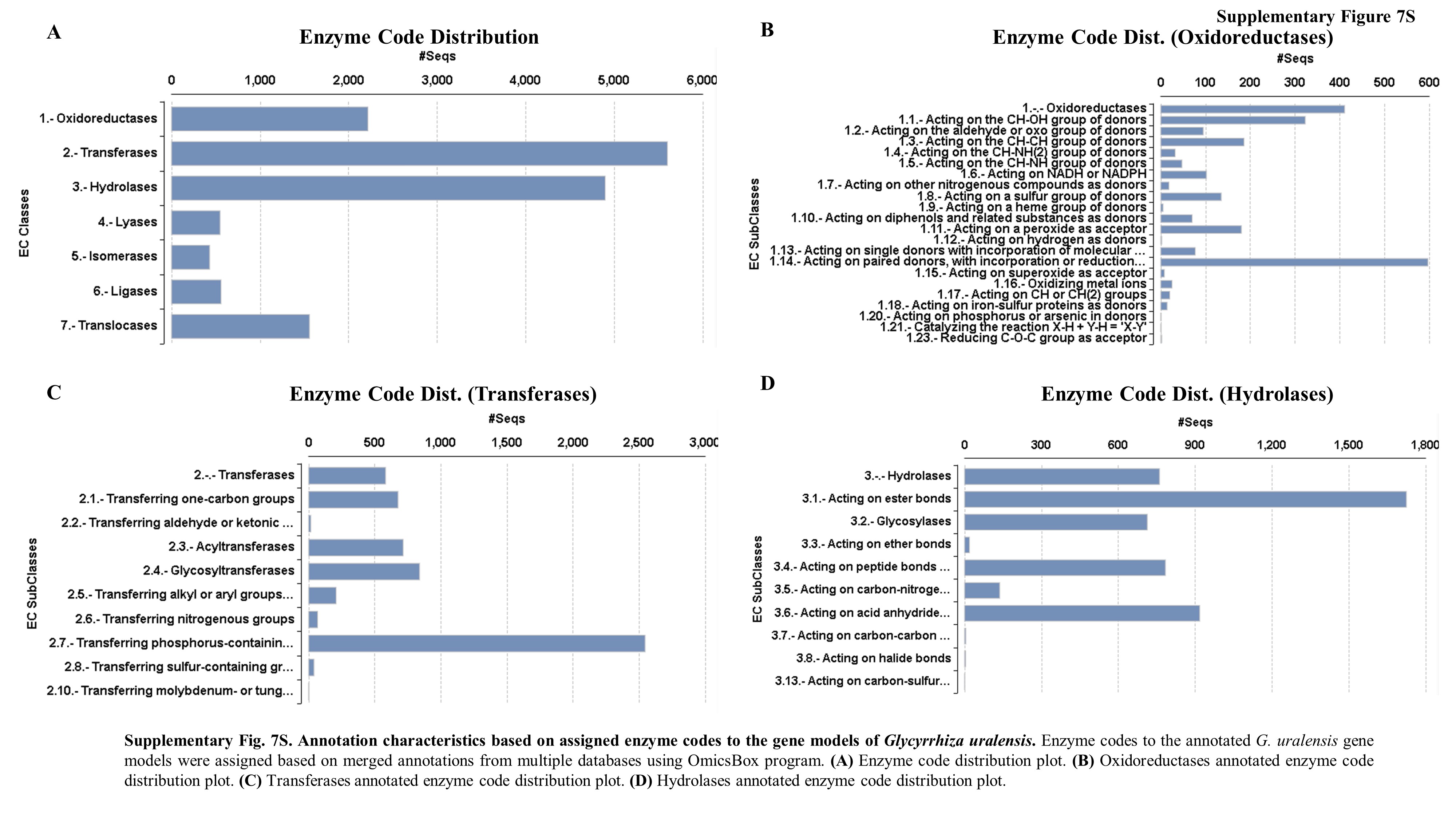

Supplement: dsac043_suppl_Supplementary_Figure_S7 [file dsac043_suppl_supplementary_figure_s7.jpeg]

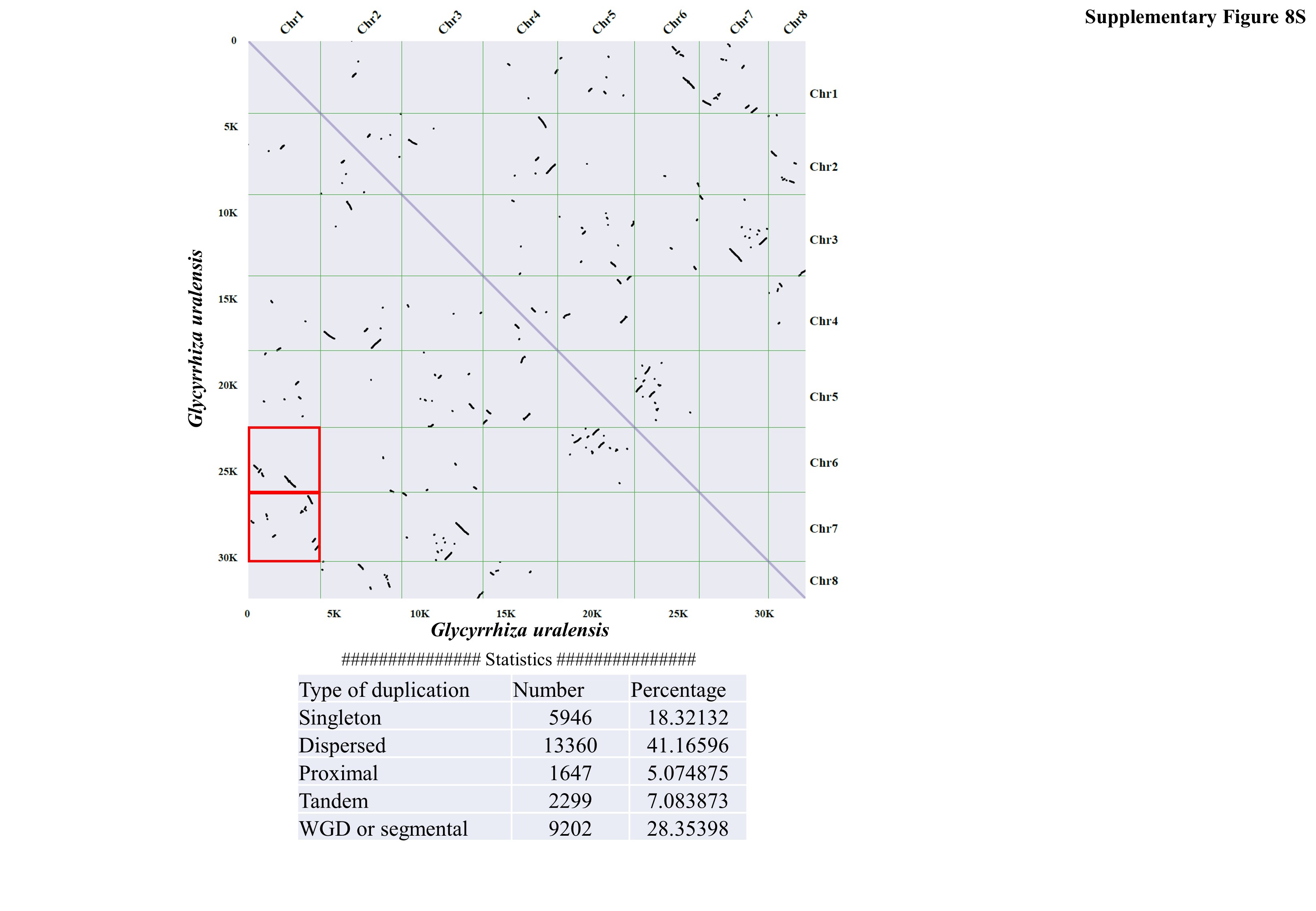

Supplement: dsac043_suppl_Supplementary_Figure_S8 [file dsac043_suppl_supplementary_figure_s8.jpeg]

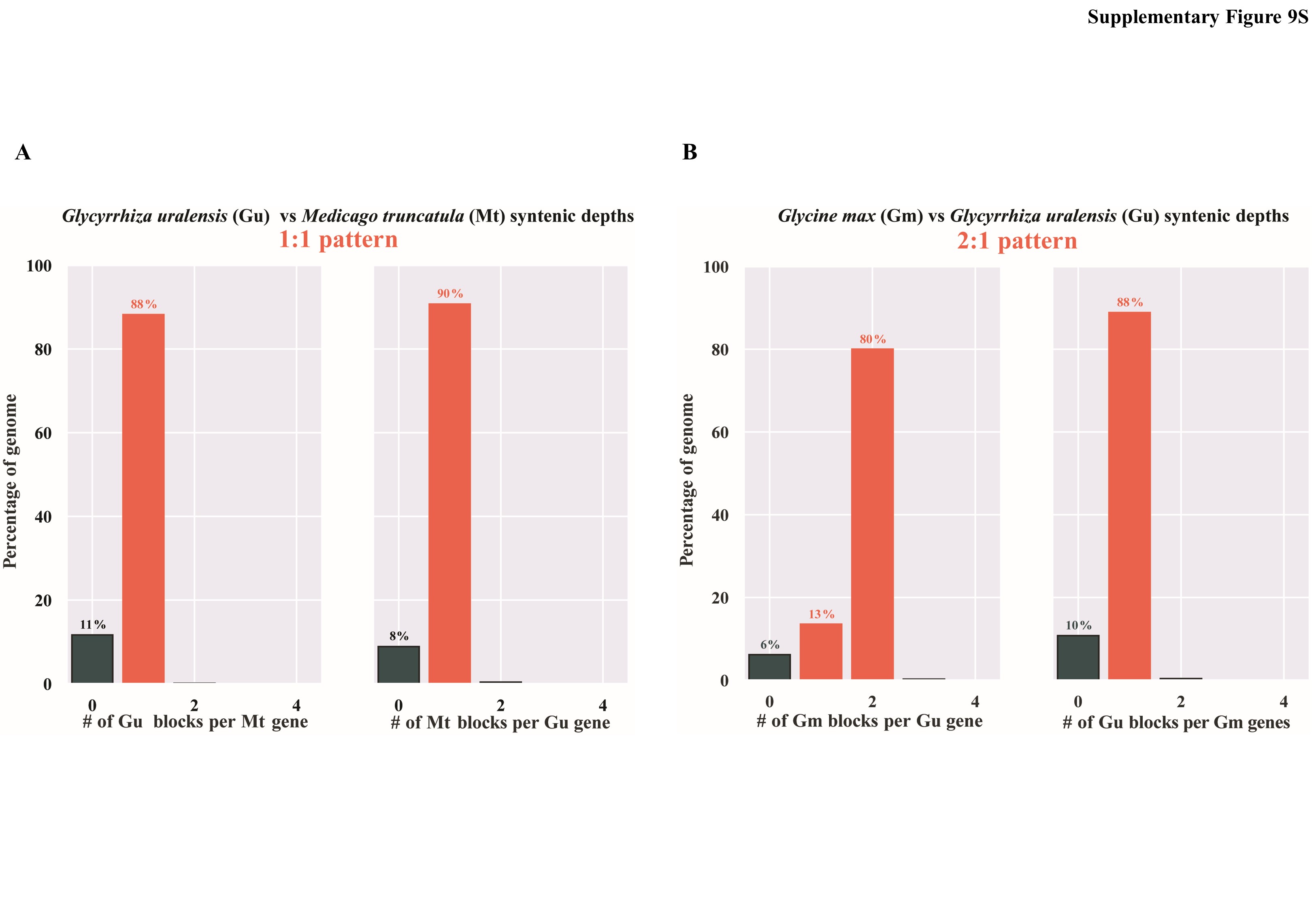

Supplement: dsac043_suppl_Supplementary_Figure_S9 [file dsac043_suppl_supplementary_figure_s9.jpeg]

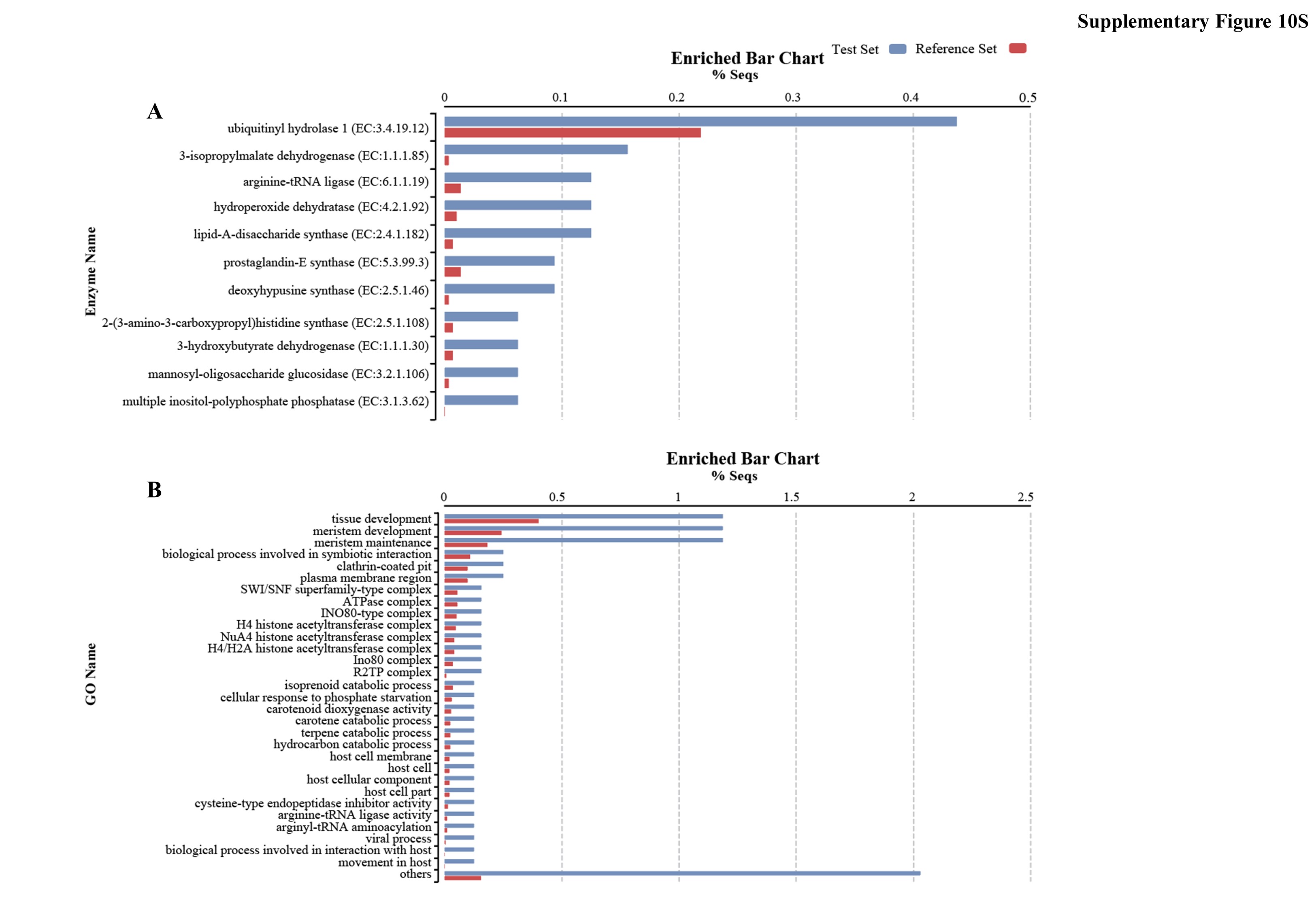

Supplement: dsac043_suppl_Supplementary_Figure_S10 [file dsac043_suppl_supplementary_figure_s10.jpeg]

# Flavonoid Biosynthesis

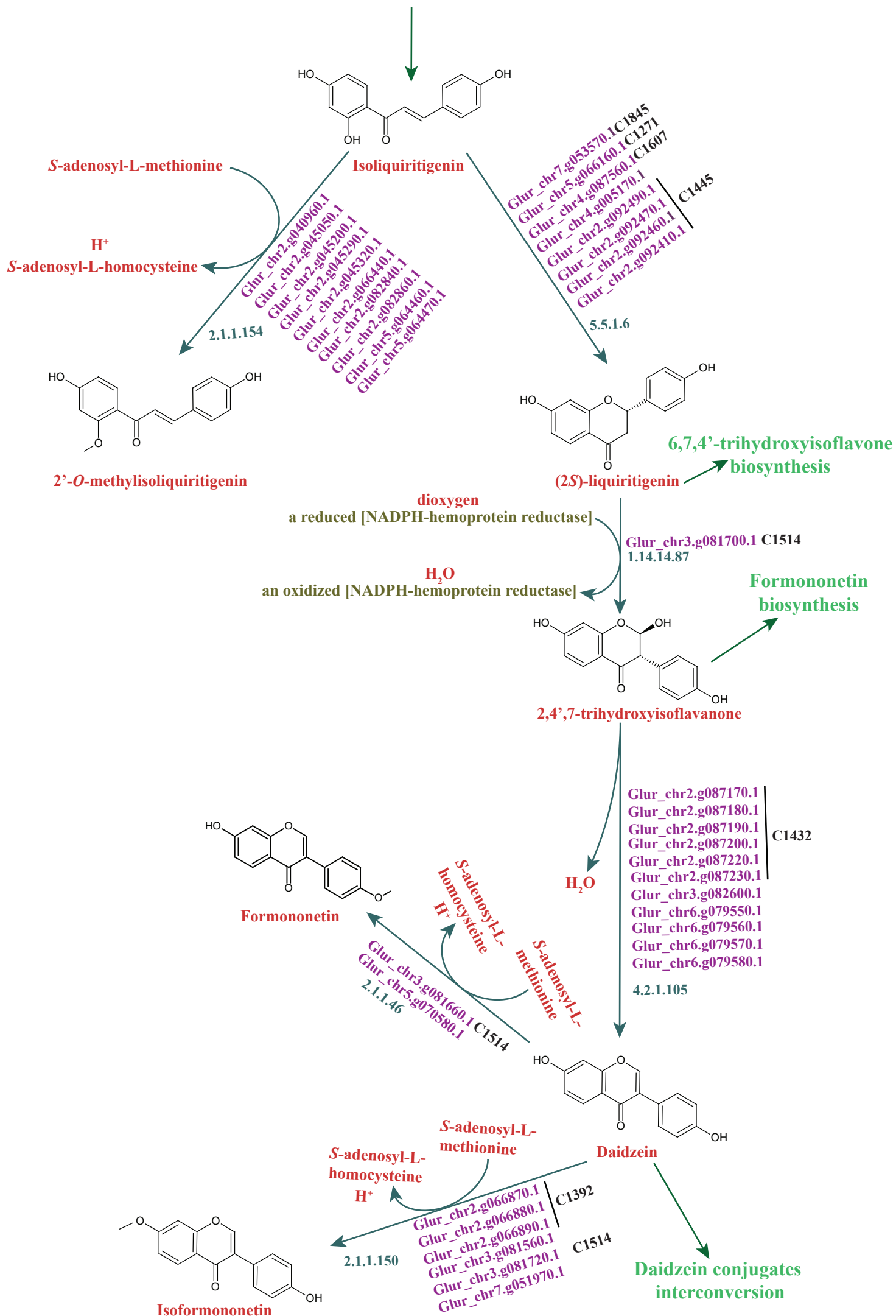

Supplement: dsac043_suppl_Supplementary_Figure_S11 [file dsac043_suppl_supplementary_figure_s11.pdf]

# Flavonoid Biosynthesis

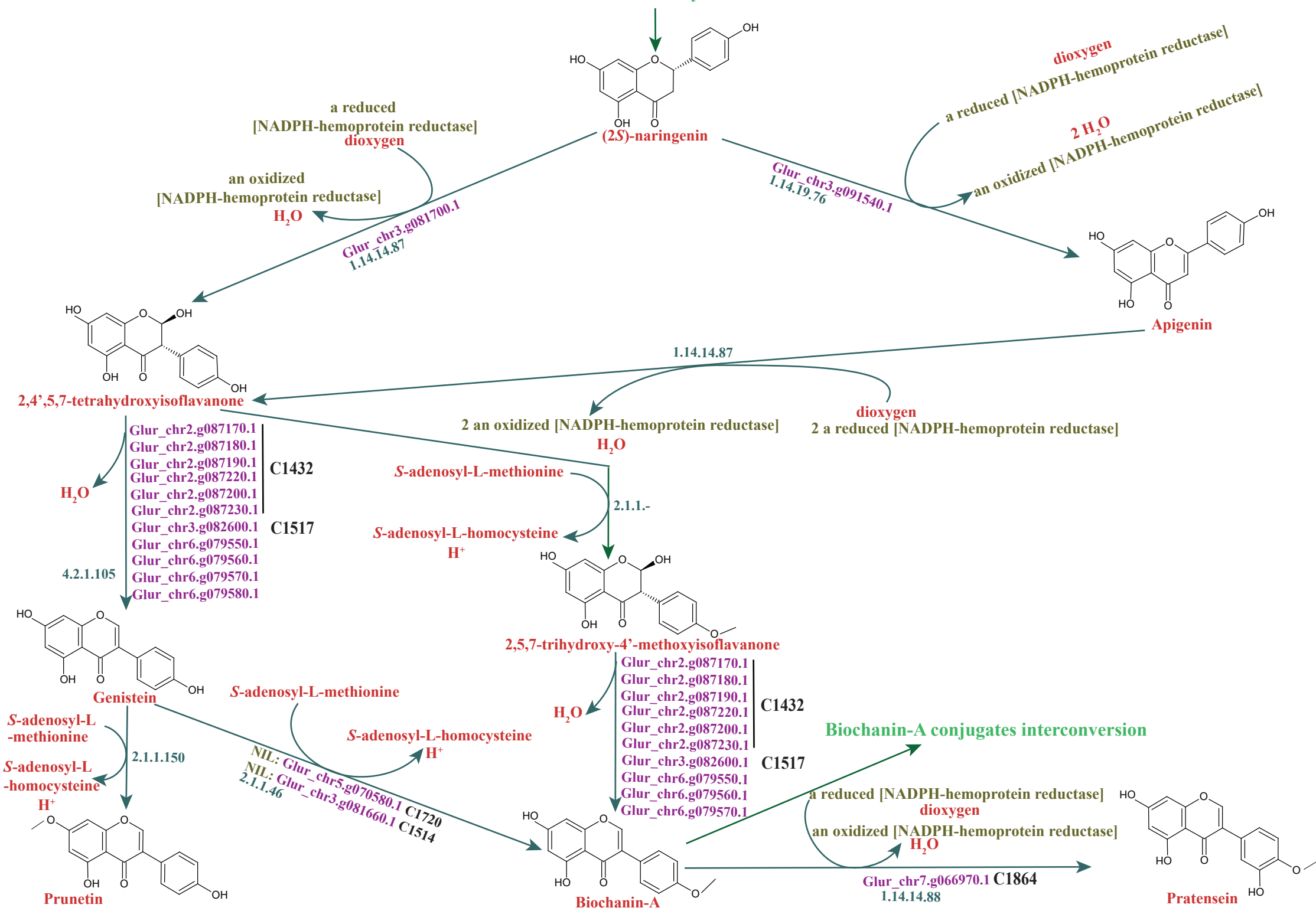

Supplement: dsac043_suppl_Supplementary_Figure_S12 [file dsac043_suppl_supplementary_figure_s12.pdf]

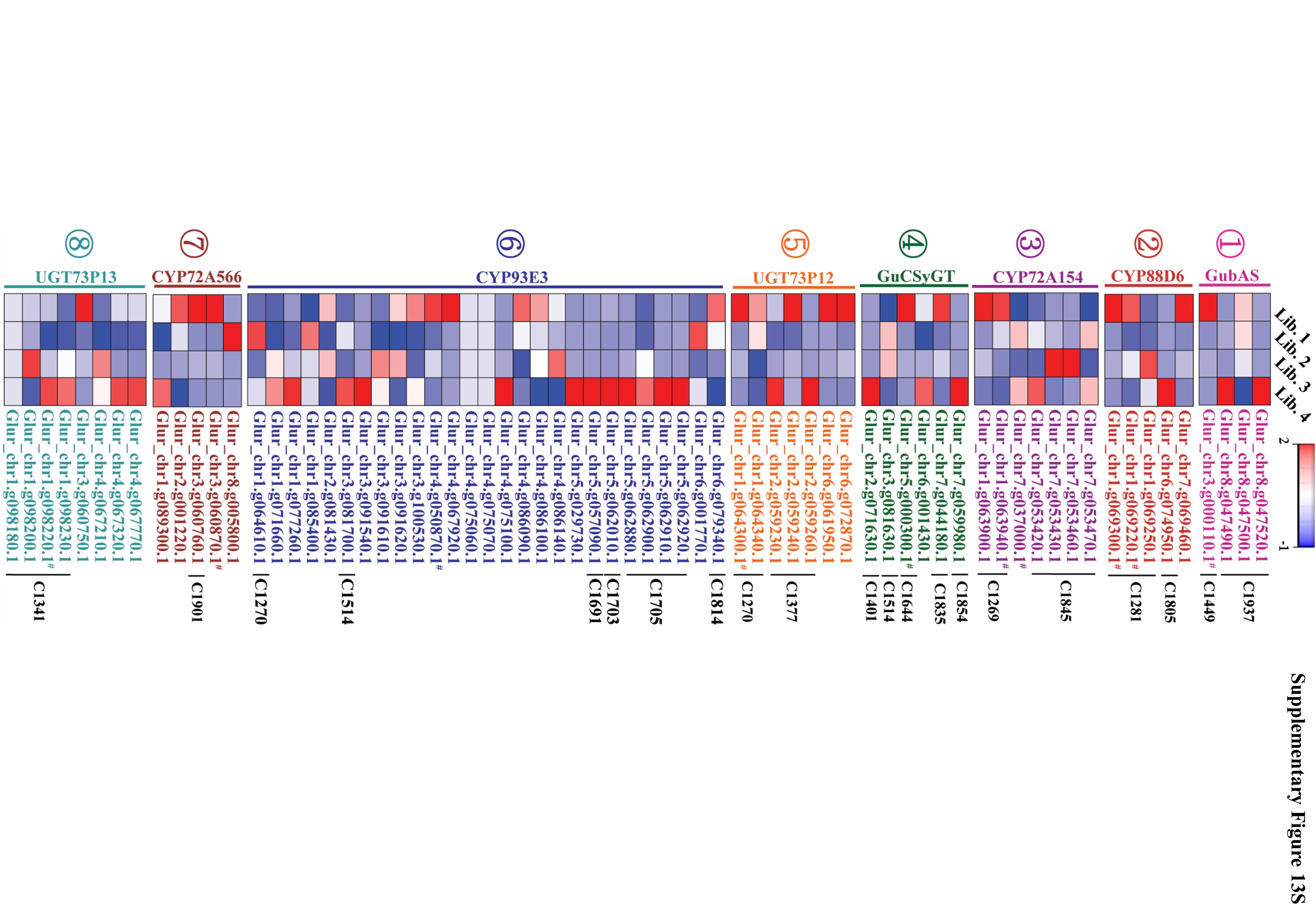

Supplement: dsac043_suppl_Supplementary_Figure_S13 [file dsac043_suppl_supplementary_figure_s13.jpeg]

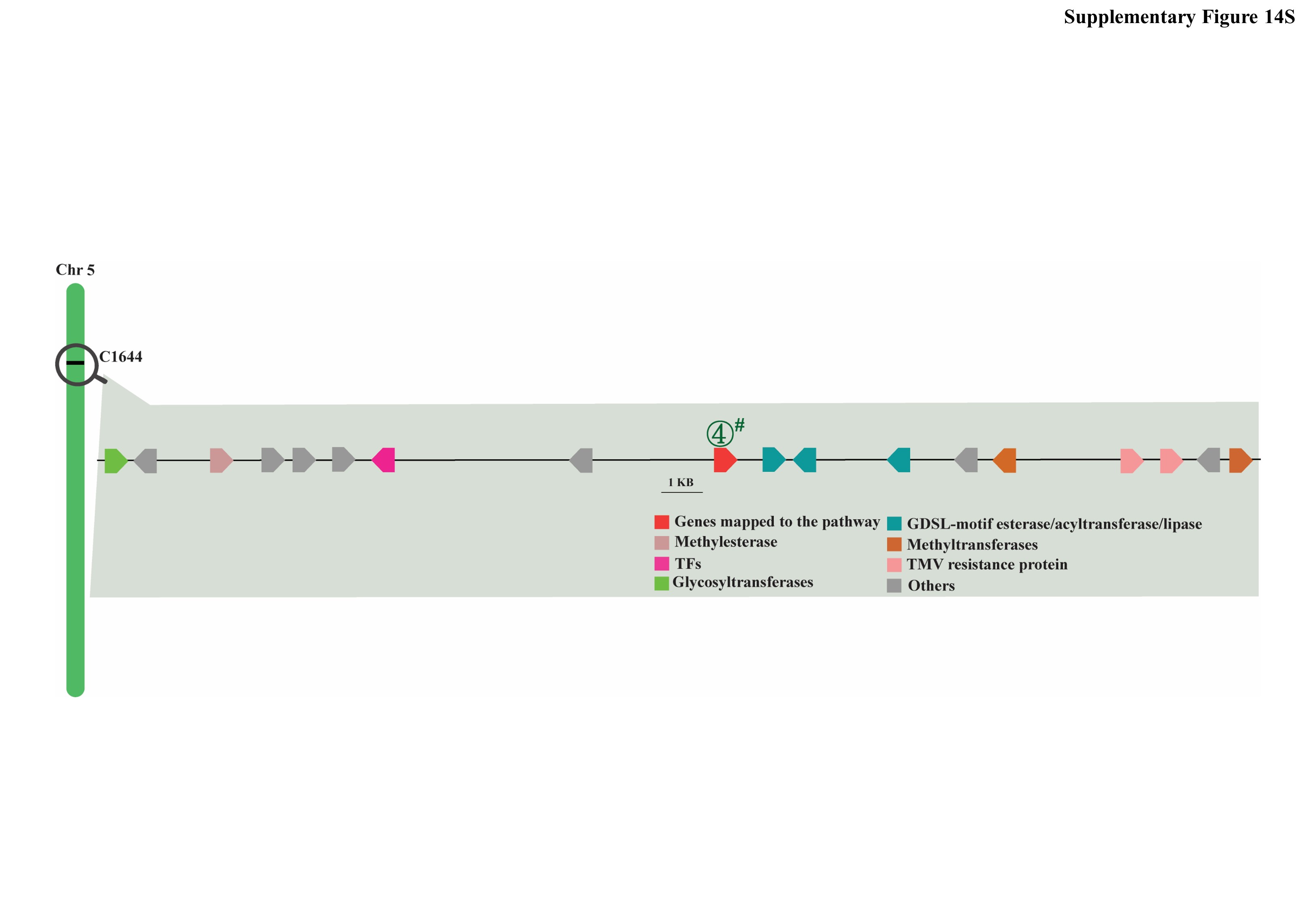

Supplement: dsac043_suppl_Supplementary_Figure_S14 [file dsac043_suppl_supplementary_figure_s14.jpeg]
