# Supplementary material for: Chromosome-scale genome assembly of Glycyrrhiza uralensis revealed metabolic gene cluster centred specialized metabolites biosynthesis
Source: DNA Res. 2022 Dec 20;29(6):dsac043. doi: 10.1093/dnares/dsac043 (PMC9763095; doi:10.1093/dnares/dsac043)
Supplement: dsac043_suppl_Supplementary_Table_S1 [file dsac043_suppl_supplementary_table_s1.docx]

**Supplementary Table S1.** The RNA-seq public datasets**^41^** used for *Glycyrrhiza uralensis* used for annotation and expression analysis

| **Sample Label** | **Run** | **BioSample** | **Bases** | **Bytes** | **Experiment** | **Sample comment** | **Sample Name** |
| --- | --- | --- | --- | --- | --- | --- | --- |
| **Gu_Lib1** | DRR006519 | SAMD00016560 | 1.17 G | 753.72 Mb | DRX005731 | Root Summer glycyrrhizin high producing strain | SAMD00016560 |
|  | DRR006520 | SAMD00016560 | 1.17 G | 765.86 Mb | DRX005731 |  | SAMD00016560 |
| **Gu_Lib2** | DRR006521 | SAMD00016563 | 1.11 G | 709.11 Mb | DRX005732 | Root Winter glycyrrhizin high producing strain | SAMD00016563 |
|  | DRR006522 | SAMD00016563 | 1.11 G | 719.38 Mb | DRX005732 |  | SAMD00016563 |
| **Gu_Lib3** | DRR006523 | SAMD00016562 | 1.17 G | 756.03 Mb | DRX005733 | Root Summer glycyrrhizin low producing strain | SAMD00016562 |
|  | DRR006524 | SAMD00016562 | 1.17 G | 765.95 Mb | DRX005733 |  | SAMD00016562 |
| **Gu_Lib4** | DRR006525 | SAMD00016561 | 1.18 G | 760.71 Mb | DRX005734 | Leaf Summer glycyrrhizin high producing strain | SAMD00016561 |
|  | DRR006526 | SAMD00016561 | 1.18 G | 771.49 Mb | DRX005734 |  | SAMD00016561 |

**^41^**Plant Cell Physiol. 2013 May;54(5):697-710. doi: 10.1093/pcp/pct057
