# Supplementary material for: Chromosome-scale genome assembly of Glycyrrhiza uralensis revealed metabolic gene cluster centred specialized metabolites biosynthesis
Source: DNA Res. 2022 Dec 20;29(6):dsac043. doi: 10.1093/dnares/dsac043 (PMC9763095; doi:10.1093/dnares/dsac043)
Supplement: dsac043_suppl_Supplementary_Table_S2 [file dsac043_suppl_supplementary_table_s2.docx]

**Supplementary Table 2S.** Assembler and parameter optimization to achieve primary contig-level genome assembly for *Glycyrrhiza uralensis*

|  | **Canu 2.2 (default parameter)** | **Falcon unzip (default parameter)** | **Hifiasm** | | | | | |
| --- | --- | --- | --- | --- | --- | --- | --- | --- |
|  |  |  | Parameter 1^#^ | Parameter 2 | Parameter 3 | Parameter 4 | Parameter 5 | Parameter 6* |
| **Assembly size (Mb)** | 878.73 | 717.33 | 487.9 | 489.25 | 491 | 482.88 | 906 | 459.30 |
| **Number of contigs** | 1137 | 979 | 386 | 395 | 421 | 391 | 3014 | 78 |
| **Largest contig length (Mb)** | 21.79 | 17.68 | 59.8 | 59.7 | 59.73 | 59.7 | 59.7 | 59.76 |
| **Smallest contig length (Kb)** | 3.69 | 27.8 | 22.4 | 21.3 | 15.23 | 21.3 | 13.1 | 17.11 |
| **Contig N50 (Mb)** | 9.78 | 11.73 | 28.95 | 28.95 | 28.94 | 29.03 | 13.8 | 32.71 |
| **Average length (Mb)** | 0.38 | 0.41 | 1.23 | 1.23 | 1.16 | 1.23 | 0.3 | 5.88 |
|  |  | | | | | | | |
| **Parameter 1#** | hifiasm -o Glur-P1.asm --h1 HiC-R1.fastq --h2 HiC-R2.fastq Hifi_reads.fastq.gz -t96 --hg-size 400m | | | | | | | |
| **Parameter 2** | hifiasm -o Glur-P4.asm --h1 HiC-R1.fastq --h2 HiC-R2.fastq Hifi_reads.fastq.gz -t96 -k60 -l3 -s 0.5 -r3 --n-weight 6 --hg-size 410m | | | | | | | |
| **Parameter 3** | hifiasm -o Glur-P4.asm --h1 HiC-R1.fastq --h2 HiC-R2.fastq Hifi_reads.fastq.gz -t96 -k45 -l3 -s 0.5 -r3 --n-weight 6 --hg-size 410m | | | | | | | |
| **Parameter 4** | hifiasm -o Glur-P4.asm --h1 HiC-R1.fastq --h2 HiC-R2.fastq Hifi_reads.fastq.gz -t96 -k39 -l3 -s 0.5 -r3 --n-weight 6 --hg-size 410m | | | | | | | |
| **Parameter 5** | hifiasm -o Glur-P5.asm --h1 HiC-R1.fastq --h2 HiC-R2.fastq Hifi_reads.fastq.gz -t96 -k39 -l3 -s 0.5 --n-weight 6 --hg-size 410m --pri-range 9 | | | | | | | |
| **Parameter 6*** | hifiasm -o Glur-P6.asm --h1 HiC-R1.fastq --h2 HiC-R2.fastq Hifi_reads.fastq.gz -t96 -k39 -l3 -s 0.5 --n-weight 6 --hg-size 410m -n 12 | | | | | | | |

* Assembly used as final contig-level genome assembly of *G. uralensis* for subsequent scaffolding; #Default parameter
