# Supplementary material for: Chromosome-scale genome assembly of Glycyrrhiza uralensis revealed metabolic gene cluster centred specialized metabolites biosynthesis
Source: DNA Res. 2022 Dec 20;29(6):dsac043. doi: 10.1093/dnares/dsac043 (PMC9763095; doi:10.1093/dnares/dsac043)
Supplement: dsac043_suppl_Supplementary_Table_S3 [file dsac043_suppl_supplementary_table_s3.docx]

**Supplementary Table 3S.** RNA-seq datasets^41^ mapping statistics to *Glycyrrhiza uralensis* genome assembly

| **Input Reads** | | **Aligned Reads** | | | | | |
| --- | --- | --- | --- | --- | --- | --- | --- |
| **Sample** | **Total Records** | **Feature** | **No Feature** | **Ambiguous** | **Alignment not Unique** | **Low Alignment Quality** | **Not aligned** |
| **Gu_Lib1** | 28,675,555 | 19,390,217 / 67.62% | 2,725,040 / 9.50% | 103,835 / 0.36% | 5,072,517 / 17.69% | 0 | 1,383,946 / 4.83% |
| **Gu_Lib2** | 26,916,556 | 18,121,637 / 67.33% | 2,850,710 / 10.59% | 84,254 / 0.31% | 4,485,039 / 16.66% | 0 | 1,374,916 / 5.11% |
| **Gu_Lib3** | 29,186,970 | 19,025,279 / 65.18% | 2,708,413 / 9.28% | 78,784 / 0.27% | 5,750,927 / 19.70% | 0 | 1,623,567 / 5.56% |
| **Gu_Lib4** | 29,272,483 | 19,097,551 / 65.24% | 2,455,984 / 8.39% | 63,108 / 0.22% | 6,669,143 / 22.78% | 0 | 986,697 / 3.37% |

**^41^**Plant Cell Physiol. 2013 May;54(5):697-710. doi: 10.1093/pcp/pct057
