# Supplementary material for: Chromosome-scale genome assembly of Glycyrrhiza uralensis revealed metabolic gene cluster centred specialized metabolites biosynthesis
Source: DNA Res. 2022 Dec 20;29(6):dsac043. doi: 10.1093/dnares/dsac043 (PMC9763095; doi:10.1093/dnares/dsac043)
Supplement: dsac043_suppl_Supplementary_Table_S4 [file dsac043_suppl_supplementary_table_s4.docx]

**Supplementary Table 4S.** The repeat classification for *Glycyrrhiza uralensis* genome assembly

|  |  |  |  |  | *Glycyrrhiza uralensis* | |
| --- | --- | --- | --- | --- | --- | --- |
|  |  |  |  |  | Length occupied (bp) | % of whole genome |
| Known repeats | Interspersed repeats | Class I | SINEs | Total | 468,672 | 0.1 |
|  |  |  | LINEs | Total | 6,259,828 | 1.4 |
|  |  |  | LTR elements | Total | 63,132,714 | 13.7 |
|  |  |  |  | Copia | 34,538,589 | 7.5 |
|  |  |  |  | Gypsy | 25,180,299 | 5.5 |
|  |  | Class II | DNA elements | | 34,834,513 | 7.6 |
|  |  | Unclassified | | | 79 | 0.0 |
|  | Helitrons | | | | 1,529,928 | 0.3 |
|  | Satellite | | | | 2,277,447 | 0.5 |
|  | Low complexity | | | | 1,287,278 | 0.3 |
|  | Simple repeat | | | | 4,649,465 | 1.0 |
|  | Unknown | | | | 33,819 | 0.0 |
|  | Subtotal | | | | 114,473,743 | 24.9 |
| Unique repeats | Unknown | | | | 168,622,954 | 36.7 |
|  | Simple repeat | | | | 28,563 | 0.0 |
|  | Subtotal | | | | 168,651,517 | 36.7 |
| Total | | | | | 283,125,260 | 61.7 |
