# Supplementary material for: Chromosome-scale genome assembly of Glycyrrhiza uralensis revealed metabolic gene cluster centred specialized metabolites biosynthesis
Source: DNA Res. 2022 Dec 20;29(6):dsac043. doi: 10.1093/dnares/dsac043 (PMC9763095; doi:10.1093/dnares/dsac043)
Supplement: dsac043_suppl_Supplementary_Table_S14 [file dsac043_suppl_supplementary_table_s14.docx]

**Supplementary Table 14S.** Reciprocal blast hit for *Glycyrrhiza uralensis* gene models and functionally characterized genes involved in the oleanane-type triterpenoid saponins

| Gene name | #A_id | B_id | A_length | B_length | A_qcovhsp | B_qcovhsp | length | pident | bitscore |
| --- | --- | --- | --- | --- | --- | --- | --- | --- | --- |
| GubAS | ACV21067.1 | Glur_chr3.g000110.1 | 762 | 763 | 100 | 99 | 762 | 99.475 | 1585 |
| CYP88D6 | BAG68929.1 | Glur_chr1.g069300.1 | 493 | 494 | 100 | 99 | 493 | 99.797 | 1021 |
| CYP72A154 | H1A988.1 | Glur_chr1.g063940.1 | 523 | 466 | 100 | 99 | 525 | 81.333 | 838 |
| GuCSyGT | BBN60794.1 | Glur_chr5.g000300.1 | 695 | 696 | 100 | 99 | 695 | 100 | 1442 |
| CYP72A566 | BBD13919.1 | Glur_chr3.g060870.1 | 528 | 519 | 98 | 99 | 518 | 99.228 | 1063 |
| UGT73P12 | BBN60804.1 | Glur_chr1.g064300.1 | 506 | 507 | 100 | 99 | 506 | 98.419 | 1029 |
| CYP93E3 | BAG68930.1 | Glur_chr4.g050870.1 | 514 | 515 | 100 | 99 | 514 | 99.805 | 1065 |
| UGT73P13 | BBN60746.1 | Glur_chr1.g098220.1 | 477 | 522 | 100 | 91 | 477 | 99.161 | 987 |
| UGT73B27 | BBN60797.1 | Glur_chr3.g083180.1 | 481 | 480 | 99 | 99 | 479 | 98.747 | 982 |
| 10GubAO/CYP88D6 | AQQ13664.1 | Glur_chr1.g069220.1 | 493 | 494 | 100 | 99 | 493 | 99.391 | 1019 |
| GmUGT72A69 | NP_001341874.1 | Glur_chr7.g037000.1 | 511 | 525 | 96 | 95 | 501 | 71.657 | 745 |

A- GenBank; B-*Glycyrrhiza uralensis* gene id
